# Causal effects of oral microbiome traits on female reproductive diseases: a two-sample Mendelian randomization study

**DOI:** 10.1186/s12905-026-04547-3

**Published:** 2026-05-22

**Authors:** Xiuye Xing, Wenjia Meng, Weiwei Chen, Xinlei Shi, Dachao Wei, Qun Lu

**Affiliations:** 1 Medical Center for Human Reproduction, Beijing Chao-Yang Hospital, Capital Medical University, 8 Gongren Tiyuchang Nanlu, Chaoyang District, Beijing, 100020 China; 2 Beijing Neurosurgical Institute, Capital Medical University, Beijing, 100070 China

**Keywords:** Two-sample Mendelian randomization, Oral microbiome traits, Genome-wide association study, Uterine leiomyoma, Tubal infertility

## Abstract

**Objective:**

Female reproductive diseases (FRDs) impose a substantial health burden. Observational studies suggest links between oral dysbiosis and systemic conditions, but whether oral microbial traits causally influence FRDs remains unclear. We used two-sample Mendelian randomization (MR) to evaluate potential causal effects of genetically predicted oral microbiome traits on FRDs.

**Methods:**

Genome-wide association study (GWAS) summary statistics for 44 salivary microbial traits were obtained from a publicly available oral microbiome GWAS based on the Danish ADDITION-PRO cohort (16 S rRNA profiling; European ancestry; *n* = 610). Outcome GWAS summary statistics for six FRDs were obtained from FinnGen (R12). The inverse-variance weighted (IVW) method was the primary analysis, complemented by MR-Egger, weighted median, and weighted mode. Sensitivity analyses included Cochran’s Q, MR-Egger intercept, MR-PRESSO, leave-one-out, and Steiger directionality tests. Multiple testing for primary IVW analyses was addressed using Benjamini–Hochberg false discovery rate (FDR) correction.

**Results:**

In primary IVW analyses, several oral taxa showed nominal associations (*P* < 0.05) with uterine leiomyoma (class *Bacilli*: OR = 1.0303, 95% CI 1.0012–1.0602; genus *Veillonella*: OR = 1.0291, 95% CI 1.0075–1.0512) and tubal infertility (family Veillonellaceae: OR = 0.8640, 95% CI 0.7824–0.9541; genus *Veillonella*: OR = 0.8900, 95% CI 0.8167–0.9699). However, none of these associations remained statistically significant after Benjamini–Hochberg FDR correction for the primary IVW analyses (all q > 0.05). In sensitivity analyses, MR-PRESSO outlier correction suggested a nominal association between *Rothia mucilaginosa* and uterine leiomyoma (OR = 1.0228, 95% CI 1.0069–1.0391; *P* = 0.0202). Overall, sensitivity analyses and Steiger directionality tests did not indicate that the main signals were driven by strong directional pleiotropy or reverse causation.

**Conclusion:**

This two-sample MR study provides suggestive, exploratory genetic evidence that specific oral microbiome traits may be linked to uterine leiomyoma and tubal infertility, but the evidence did not remain statistically significant after multiple-testing correction. Larger oral microbiome GWAS, independent outcome datasets, and functional studies are needed to validate these signals and clarify biological mechanisms.

**Supplementary Information:**

The online version contains supplementary material available at 10.1186/s12905-026-04547-3.

## Introduction

 The ovaries and uterus are essential reproductive and endocrine organs. Female reproductive diseases (FRDs)—including uterine leiomyoma, endometriosis, polycystic ovary syndrome (PCOS), premature ovarian insufficiency, pelvic inflammatory sequelae, and infertility—affect quality of life and contribute to a substantial clinical and societal burden [1]. Inflammatory signaling and endocrine dysregulation (notably estrogen and progesterone pathways) are implicated in the pathogenesis of several FRDs, while tubal infertility is frequently related to chronic pelvic inflammation and infection-driven tissue damage [2–4].

Accumulating evidence suggests that disturbances in the oral microbial community (oral dysbiosis) are linked to systemic diseases beyond the oral cavity [5, 6]. Oral inflammation and dysbiosis may promote low-grade systemic inflammation through transient bacteremia and circulation of microbial components and metabolites. For example, lipopolysaccharide (LPS) can activate Toll-like receptor 4 (TLR4) on innate immune cells (e.g., monocytes/macrophages), promoting cytokine release such as IL-6, IL-1β, and TNF-α and increasing inflammatory biomarkers (e.g., C-reactive protein) [7]. Microbial metabolites, including short-chain fatty acids (SCFAs), can signal via G-protein–coupled receptors (e.g., FFAR2/GPR43 and FFAR3/GPR41) and may also modulate histone deacetylase activity [8, 9]. These systemic immune and metabolic changes may intersect with endocrine regulation via the hypothalamic–pituitary–gonadal axis and tissue steroid responsiveness, offering plausible routes by which oral dysbiosis could influence reproductive health. Nevertheless, observational associations are vulnerable to confounding (e.g., socioeconomic status, smoking, obesity, and health behaviors) and reverse causation. In addition to microbial components and metabolites, oral bacteria can produce other bioactive molecules such as bacteriocins, which may contribute to host–microbe interactions [10]. Systemic immunometabolic regulation in immune cells, including solute carrier transporters, provides another interface by which microbial signals may shape inflammatory responses [11]. Furthermore, oral streptococcal infections have been linked to autoimmune phenomena, and trained immunity has been implicated in autoimmune responses [12, 13]. Evidence from pregnancy research also suggests that placental Toll-like receptor recognition of salivary and subgingival microbiota is associated with pregnancy complications [14]. Recent reviews further highlight age-related oral dysbiosis and systemic comorbidities, the dynamic host interactions of the oral microbiota, and potential viral contributions in periodontitis [15–17].

A growing body of research has explored microbiome–reproductive links, mostly focusing on gut, vaginal, and endometrial microbiota [18–21]. Compared with these niches, the oral microbiome is an accessible microbial ecosystem and a potential upstream contributor to systemic immune and metabolic states [5, 6]. However, whether variation in oral microbial traits has a causal role in FRDs remains unclear.

Mendelian randomization (MR) is a genetic epidemiologic approach that uses germline variants associated with an exposure as instrumental variables to estimate the causal effect of that exposure on an outcome, mitigating confounding and reverse causation under key assumptions [22–24]. Because MR estimates the effect of genetically predicted microbial traits rather than the direct effect of microbial exposure itself, interpretation should be framed at the level of host genetic determinants of microbial composition and their downstream consequences.

In this study, we performed a two-sample MR analysis to evaluate the causal effects of oral microbiome traits on six FRDs using publicly available GWAS summary statistics. We further applied extensive sensitivity analyses and multiple-testing correction to provide a transparent and conservative assessment of the evidence.

## Methods

### Study design

**Fig. 1:**
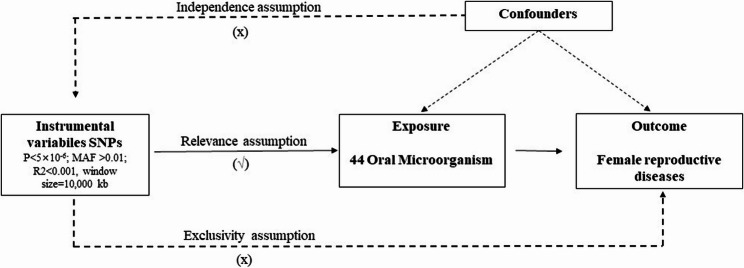
Overview of Mendelian randomization

The MR analysis followed the Strengthening the Reporting of Observational Studies in Epidemiology using Mendelian Randomization (STROBE-MR) guidelines [25]. Our MR analysis was conducted based on three core instrumental variable (IV) assumptions [26]: (1) relevance, the genetic instruments are robustly associated with the exposure; (2) independence, the instruments are not associated with confounders of the exposure–outcome relationship; and (3) exclusion restriction, the instruments influence the outcome only through the exposure and not via alternative pathways (i.e., no horizontal pleiotropy). A study framework diagram is provided in Fig. 1 to depict the design of our study. As all GWAS summary statistics used in this study were publicly available and de-identified, no additional ethical approvals were required.

### Data source

The outcome GWAS summary dataset for uterine leiomyoma, polycystic ovary syndrome, endometriosis, and tubal infertility were obtained from FinnGen (Table 1). Exposure GWAS summary statistics for oral microbiome traits were obtained from Stankevic et al., who conducted GWAS of salivary microbiota in 610 unrelated individuals from the Danish ADDITION-PRO cohort using 16 S rRNA gene amplicon sequencing (Table 1). The exposure dataset comprised 44 microbial traits (including taxon abundance traits across different taxonomic levels and one beta-diversity trait based on Bray–Curtis dissimilarity), with association models adjusted for key demographic, lifestyle, and technical covariates as described in the original study. All data used were publicly available, de-identified, and derived from studies with appropriate ethical approval and participant consent [27].

**Table 1:** The GWAS data for exposure and outcomes

Trait	GWAS ID	Population	
		case/control	Decent
Leiomyoma of uterus	CD2_BENIGN_LEIOMYOMA_UTERI	42,107/239,957	European
Polycystic ovarian syndrome	E4_PCOS	2,214/267,780	European
Endometriosis	N14_ENDOMETRIOSIS	20,190/130,160	European
Female infertility, tubal origin	N14_FITUB	2,038/130,160	European
Habitual aborter	N14_HABITABORT	811/130,160	European
Spontaneous abortion	O15_ABORT_SPONTAN	23,167/199,279	European
Saliva microbiota abundance (Phylum Firmicutes)	GCST90429799	610	European
Saliva microbiota abundance (Phylum Proteobacteria)	GCST90429800	610	European
Saliva microbiota abundance (Class *Bacilli*)	GCST90429801	610	European
Saliva microbiota abundance (Order Bacteroidales)	GCST90429802	610	European
Saliva microbiota abundance (Order Fusobacteriales)	GCST90429803	610	European
Saliva microbiota abundance (Order Actinomycetales)	GCST90429804	610	European
Saliva microbiota abundance (Order Clostridiales)	GCST90429805	610	European
Saliva microbiota abundance (Family Veillonellaceae)	GCST90429806	610	European
Saliva microbiota abundance (Family Pasteurellaceae)	GCST90429807	610	European
Saliva microbiota abundance (Family Prevotellaceae)	GCST90429808	610	European
Saliva microbiota abundance (Family Actinomycetaceae)	GCST90429809	610	European
Saliva microbiota abundance (Family Lachnospiraceae_[XIV])	GCST90429810	610	European
Saliva microbiota abundance (Genus *Veillonella*)	GCST90429811	610	European
Saliva microbiota abundance (Genus *Haemophilus*)	GCST90429812	610	European
Saliva microbiota abundance (Genus *Streptococcus*)	GCST90429813	610	European
Saliva microbiota abundance (Genus *Neisseria*)	GCST90429814	610	European
Saliva microbiota abundance (Genus *Prevotella*)	GCST90429815	610	European
Saliva microbiota abundance (Genus *Porphyromonas*)	GCST90429816	610	European
Saliva microbiota abundance (Genus *Fusobacterium*)	GCST90429817	610	European
Saliva microbiota abundance (Genus *Rothia*)	GCST90429818	610	European
Saliva microbiota abundance (Genus *Schaalia*)	GCST90429819	610	European
Saliva microbiota abundance (Genus *Granulicatella*)	GCST90429820	610	European
Saliva microbiota abundance (Genus *Leptotrichia*)	GCST90429821	610	European
Saliva microbiota abundance (Genus *Alloprevotella*)	GCST90429822	610	European
Saliva microbiota abundance (unknown Veillonella species (ASV0001))	GCST90429823	610	European
Saliva microbiota abundance (Species parainfluenzae)	GCST90429824	610	European
Saliva microbiota abundance (unknown Streptococcus species (ASV0003))	GCST90429825	610	European
Saliva microbiota abundance (unknown Neisseria species (ASV0004))	GCST90429826	610	European
Saliva microbiota abundance (Species histicola)	GCST90429827	610	European
Saliva microbiota abundance (unknown Streptococcus species (ASV0006))	GCST90429828	610	European
Saliva microbiota abundance (Species parvula)	GCST90429829	610	European
Saliva microbiota abundance (unknown Porphyromonas species (ASV0008))	GCST90429830	610	European
Saliva microbiota abundance (unknown Streptococcus species (ASV0009))	GCST90429831	610	European
Saliva microbiota abundance (Species periodonticum)	GCST90429832	610	European
Saliva microbiota abundance (Species dispar)	GCST90429833	610	European
Saliva microbiota abundance (unknown Rothia species (ASV0012))	GCST90429834	610	European
Saliva microbiota abundance (Species micronuciformis)	GCST90429835	610	European
Saliva microbiota abundance (Species pallens)	GCST90429836	610	European
Saliva microbiota abundance (*Rothia mucilaginosa*)	GCST90429837	610	European
Saliva microbiota abundance (unknown Rothia species (ASV0016))	GCST90429838	610	European
Saliva microbiota abundance (unknown Schaalia species (ASV0017))	GCST90429839	610	European
Saliva microbiota abundance (Species rogosae)	GCST90429840	610	European
Saliva microbiota abundance (unknown Gemella)	GCST90429841	610	European
Beta diversity of salivary microbiota	GCST90429842	610	European

### IVs selection

Single nucleotide polymorphisms (SNPs) associated with each oral microbiome trait were used as instrumental variables (IVs) for Mendelian randomization (MR). SNPs were selected at a significance threshold of *P* < 5 × 10⁻⁶ to ensure sufficient instrument availability given the modest sample size of the exposure GWAS, a strategy commonly adopted in microbiome MR studies [28]. We further filtered SNPs with minor allele frequency (MAF) > 0.01. To obtain independent instruments, linkage disequilibrium (LD) clumping was performed using r² < 0.001 within a 10,000 kb window. Palindromic SNPs with ambiguous strand orientation were excluded. When an IV was not available in the outcome GWAS, a proxy SNP in high LD (r² > 0.8) was used [29].

Instrument strength was evaluated using the F-statistic to reduce weak instrument bias. For each SNP, the proportion of exposure variance explained (R²) was calculated as: R² = 2 × EAF × (1 − EAF) × β², where EAF is the effect allele frequency and β is the SNP–exposure effect estimate. The F-statistic was then calculated as: F = R² × (*N* − 2) / (1 − R²), where N denotes the sample size of the exposure GWAS (*N* = 610). SNPs with F ≤ 10 were considered weak instruments and were excluded [30].

To further minimize confounding, all candidate instruments were queried in the GWAS Catalog to identify SNPs associated with potential confounders or the outcomes themselves. Confounders of interest included body mass index/obesity, smoking, alcohol consumption, glycaemic traits/type 2 diabetes, and inflammatory markers (e.g., C-reactive protein). MR analyses were repeated after excluding such SNPs.

### MR analysis

Inverse-variance weighted (IVW) MR was used as the primary method to estimate causal effects [31]. Given the potential for heterogeneity across SNP-specific Wald ratios, we used a multiplicative random-effects IVW model as appropriate. Complementary methods (MR-Egger, weighted median, and weighted mode) were used for robustness [32]. To account for multiple comparisons across the set of tested exposure–outcome pairs, Benjamini–Hochberg FDR correction was applied to IVW results; associations with q < 0.05 were considered statistically significant, and *P* < 0.05 was considered nominal. Statistical power for the nominally significant MR estimates was calculated using the mRnd online calculator (https://shiny.cnsgenomics.com/mRnd/), incorporating the sample size of the outcome GWAS, the proportion of variance in the exposure explained by the IVs (R2), and the observed odds ratio, with a Type-I error rate (α) set at 0.05 [33].

### Sensitivity analysis

Sensitivity analyses were performed to assess heterogeneity and horizontal pleiotropy. Cochran’s Q statistic was used to evaluate heterogeneity [34], and the MR-Egger intercept test was used to detect directional pleiotropy [35]. MR-PRESSO was applied to identify potential outlier SNPs and provide outlier-corrected estimates [36]. Steiger directionality tests were conducted to assess whether the instruments explained more variance in the exposure than in the outcome, supporting the assumed causal direction [37].

## Results

### IVs selection

In total, GWAS summary statistics were available for 44 oral microbiome traits. After instrument selection and harmonization with outcome data, not all traits yielded eligible instruments for every outcome; therefore, the primary IVW analyses included 246 analyzable exposure–outcome pairs. Instrument strength metrics indicated generally adequate instrument strength (F-statistics > 10; Tables S1–S2).

### Causal association between oral microbiome and FRDs

**Fig. 2:**
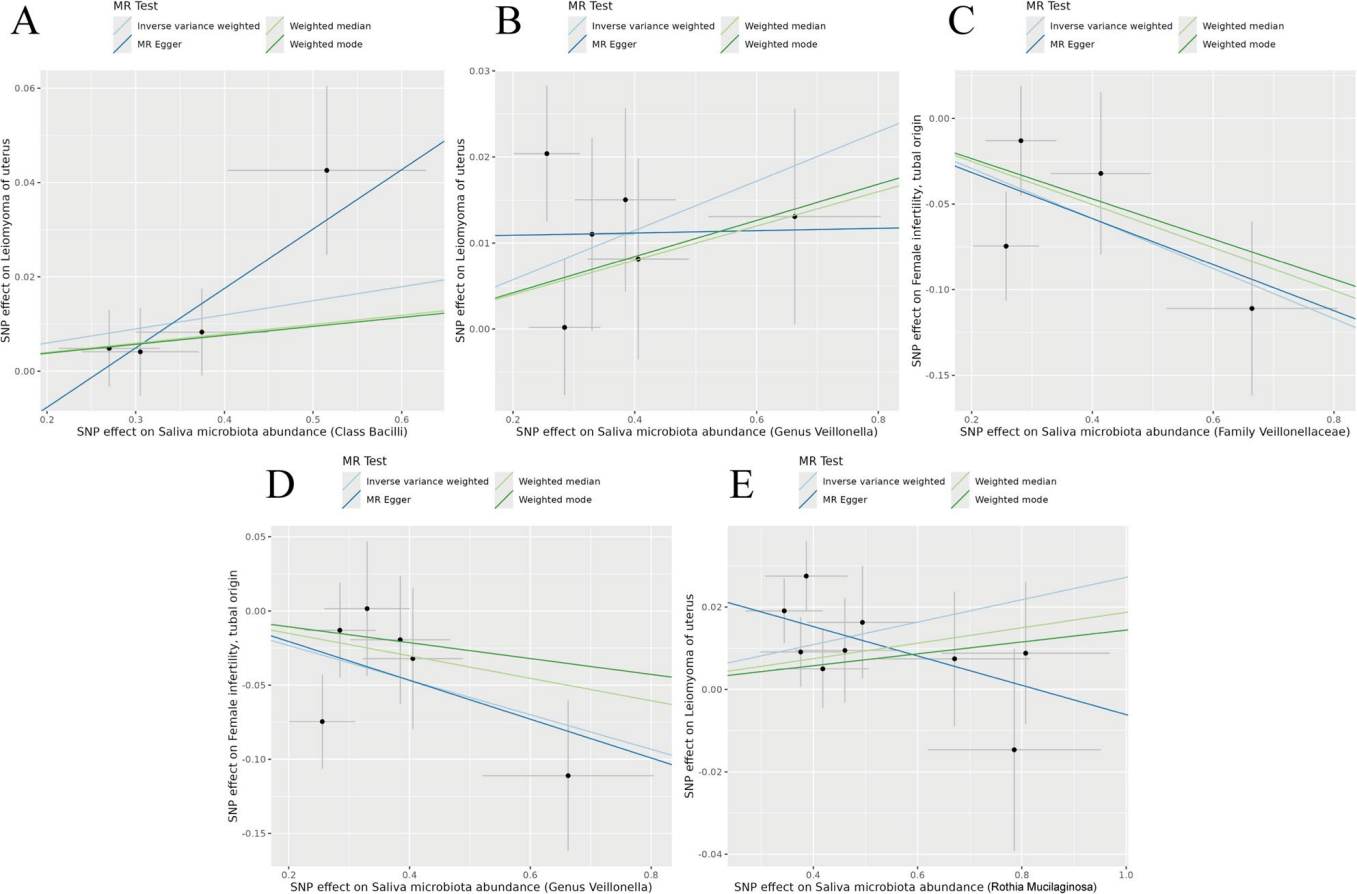
Scatter plots showing the causal associations between oral microbiota and female reproductive diseases. **A**, **B**: The plots of class *Bacilli* and genus *Veillonella* on uterine leiomyoma; (**C**, **D**): The plots of family Veillonellaceae and genus *Veillonella* on female infertility of tubal origin; (**E**): The plot of *Rothia mucilaginosa* on uterine leiomyoma. Each point represents a Single nucleotide polymorphism (SNP) serving as an instrumental variable for the exposure variable. The regression line’s slope, derived via inverse variance weighting and MR-Egger methods, denotes the estimated causal effect. The horizontal and vertical axes correspond respectively to the genetic association between the exposure variable (oral microbiota abundance) and the outcome variable (risk of female reproductive disorders). The shaded regions denote the 95% confidence intervals for the regression lines

**Fig. 3:**
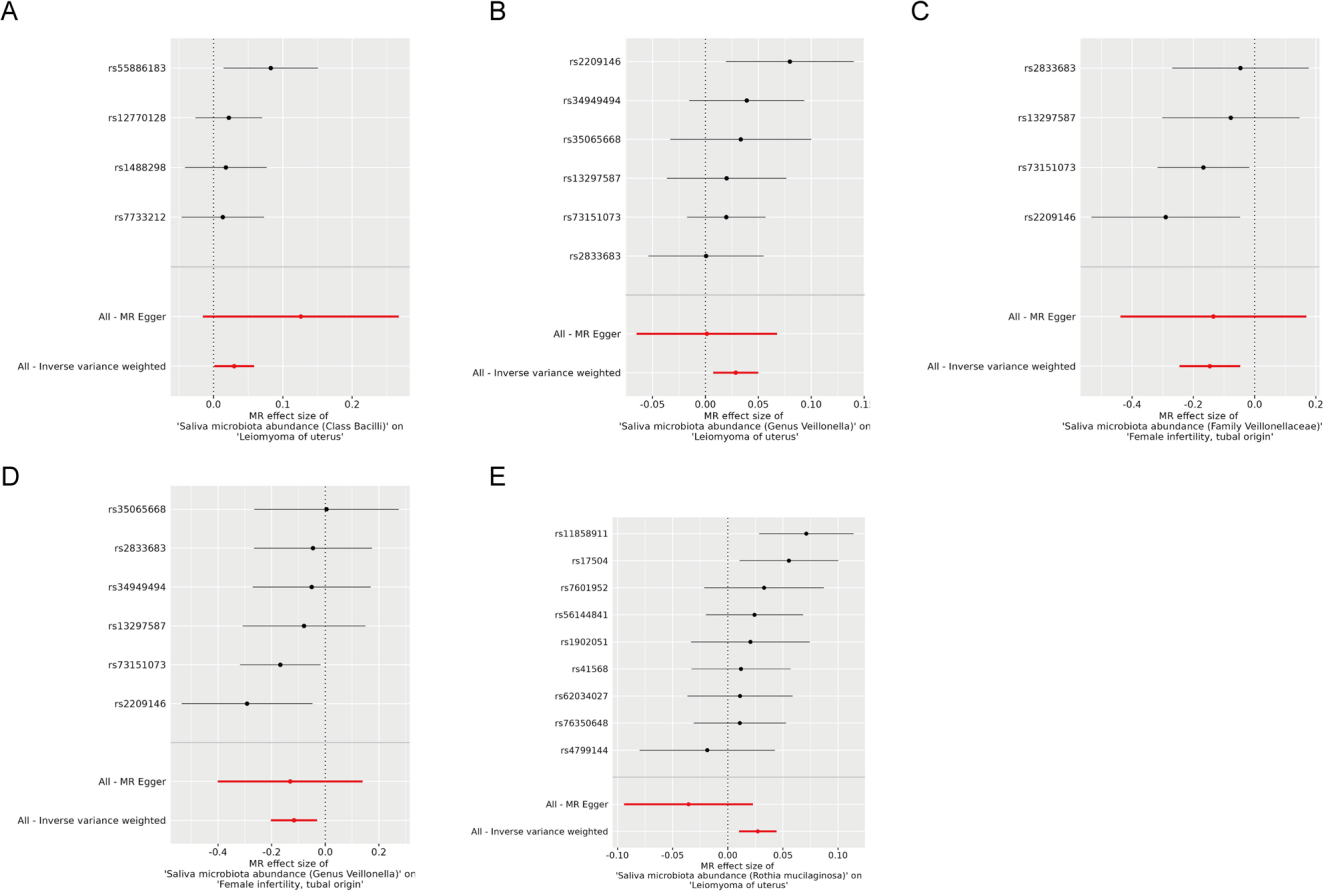
Forest plots of the causal associations between oral microbiota and female reproductive diseases. **A**, **B**: The plots of class *Bacilli* and genus *Veillonella* on uterine leiomyoma; (**C**, **D**): The plots of family Veillonellaceae and genus *Veillonella* on female infertility of tubal origin; (**E**): The plot of *Rothia mucilaginosa* on uterine leiomyoma. Odds ratios (ORs) and 95% Confidence intervals (CIs) were estimated using the Inverse-variance weighted (IVW) method. The size of each square reflects the weight of the corresponding SNP in the meta-analysis. Horizontal lines represent 95% CIs. The diamond at the bottom represents the pooled causal estimate

In IVW analyses, nominal associations were observed between class *Bacilli* and uterine leiomyoma (OR = 1.030, 95%CI: 1.001–1.060, *P* = 0.041), as well as between genus *Veillonella* and uterine leiomyoma (OR = 1.029, 95% CI: 1.007–1.051, *P* = 0.008). Conversely, nominal protective associations against tubal infertility were observed for family Veillonellaceae (OR = 0.864, 95% CI: 0.782–0.954, *P* = 0.004) and genus *Veillonella* (OR = 0.890, 95% CI: 0.816–0.969, *P* = 0.008) (Table 2). The individual SNP effects and pooled causal estimates for these nominal associations are visualized in scatter plots (Fig. 2A-D) and forest plots (Fig. 3A-D). However, after Benjamini–Hochberg FDR correction for the primary IVW analyses, none of the associations met the q < 0.05 threshold, and thus these findings should be interpreted as suggestive rather than definitive (Table 2).

**Table 2:** Relationship between oral microbiota and FRDs (nominal IVW results; BH-FDR q values for primary IVW analyses)

Exposure	Outcome	*N*.SNP	Method	OR (95% CI)	*P*	BH-FDR q
Saliva microbiota abundance (Class *Bacilli*)	Leiomyoma of uterus	4	Inverse variance weighted	1.0303 (1.0012–1.0602)	0.0413	0.75645
Saliva microbiota abundance (Family Veillonellaceae)	Female infertility, tubal origin	4	Inverse variance weighted	0.864 (0.7824–0.9541)	0.0039	0.15990
Saliva microbiota abundance (Genus *Veillonella*)	Female infertility, tubal origin	6	Inverse variance weighted	0.89 (0.8167–0.9699)	0.0079	0.16195
Saliva microbiota abundance (Genus *Veillonella*)	Leiomyoma of uterus	6	Inverse variance weighted	1.0291 (1.0075–1.0512)	0.0081	0.33210

Sensitivity analyses were conducted to assess heterogeneity and horizontal pleiotropy. Cochran’s Q test and the MR-Egger intercept were used to evaluate heterogeneity and directional pleiotropy, respectively (Table S3). MR-PRESSO was applied to identify potential outlier SNPs and to obtain outlier-corrected estimates when outliers were detected (Table S4), and the corresponding outlier-removed results are provided in Table S5.

MR-PRESSO identified outlier SNPs in a subset of exposure–outcome pairs. After outlier correction, a nominal association between the species *Rothia mucilaginosa* and uterine leiomyoma was observed (OR = 1.0228, 95% CI 1.0069–1.0391; *P* = 0.0202; outlier SNP: rs953559; Table S4). Across the full set of analyses, no associations remained significant after FDR correction, and the overall pattern should be interpreted as exploratory. In addition to the primary IVW results, several nominal associations were observed in the weighted median analyses; however, these did not remain significant after multiple-testing correction and were not consistently supported across complementary MR methods (Table S6). Steiger directionality testing supported the assumed direction from oral microbiome traits to FRDs in the analyzable pairs (Table S8).

**Fig. 4:**
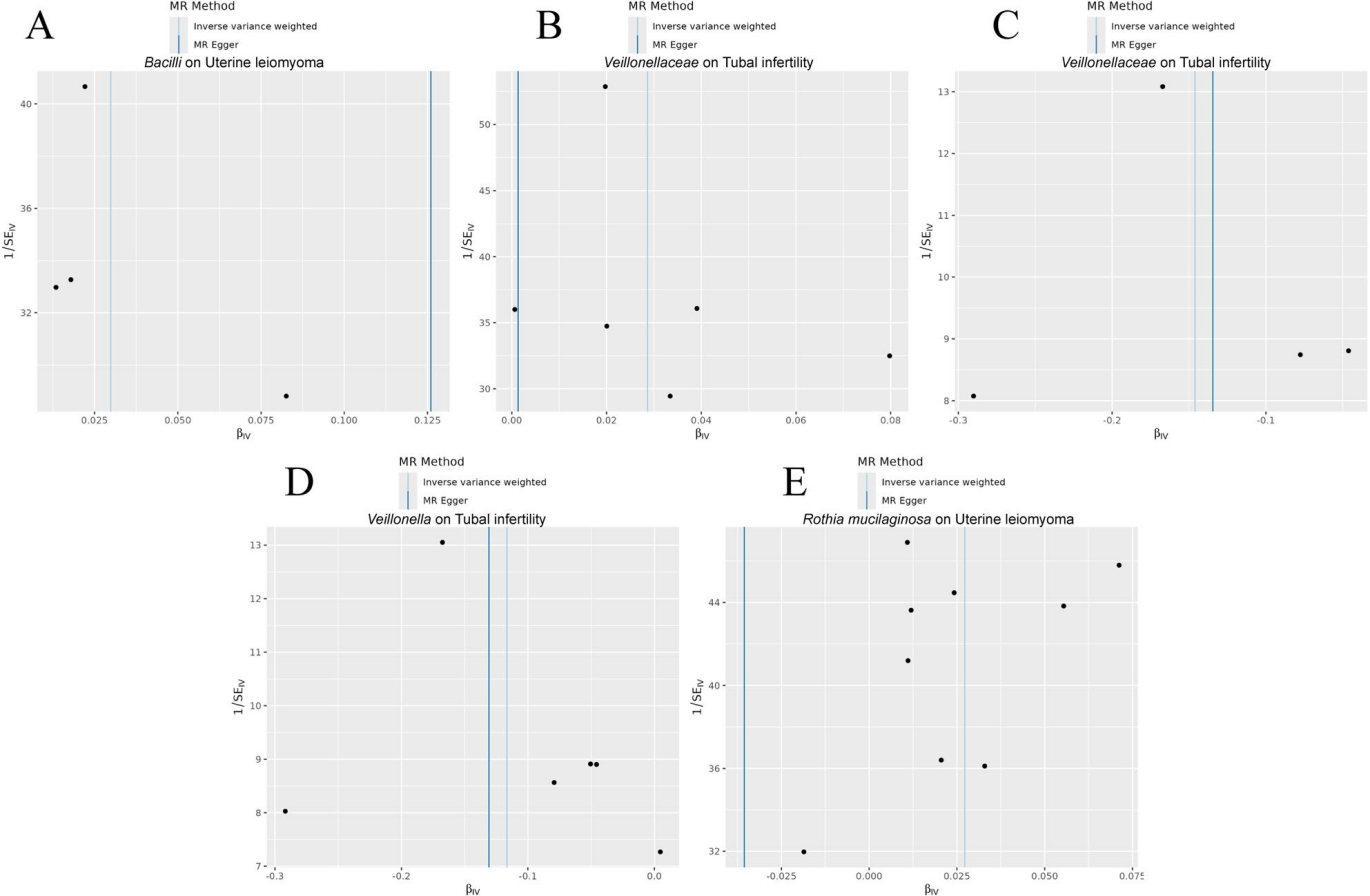
Funnel plots of the causal associations between oral microbiota and female reproductive diseases. **A**, **B**: The plots of class *Bacilli* and genus *Veillonella* on uterine leiomyoma; (**C**, **D**): The plots of family Veillonellaceae and genus *Veillonella* on female infertility of tubal origin; (**E**): The plot of *Rothia mucilaginosa* on uterine leiomyoma

**Fig. 5:**
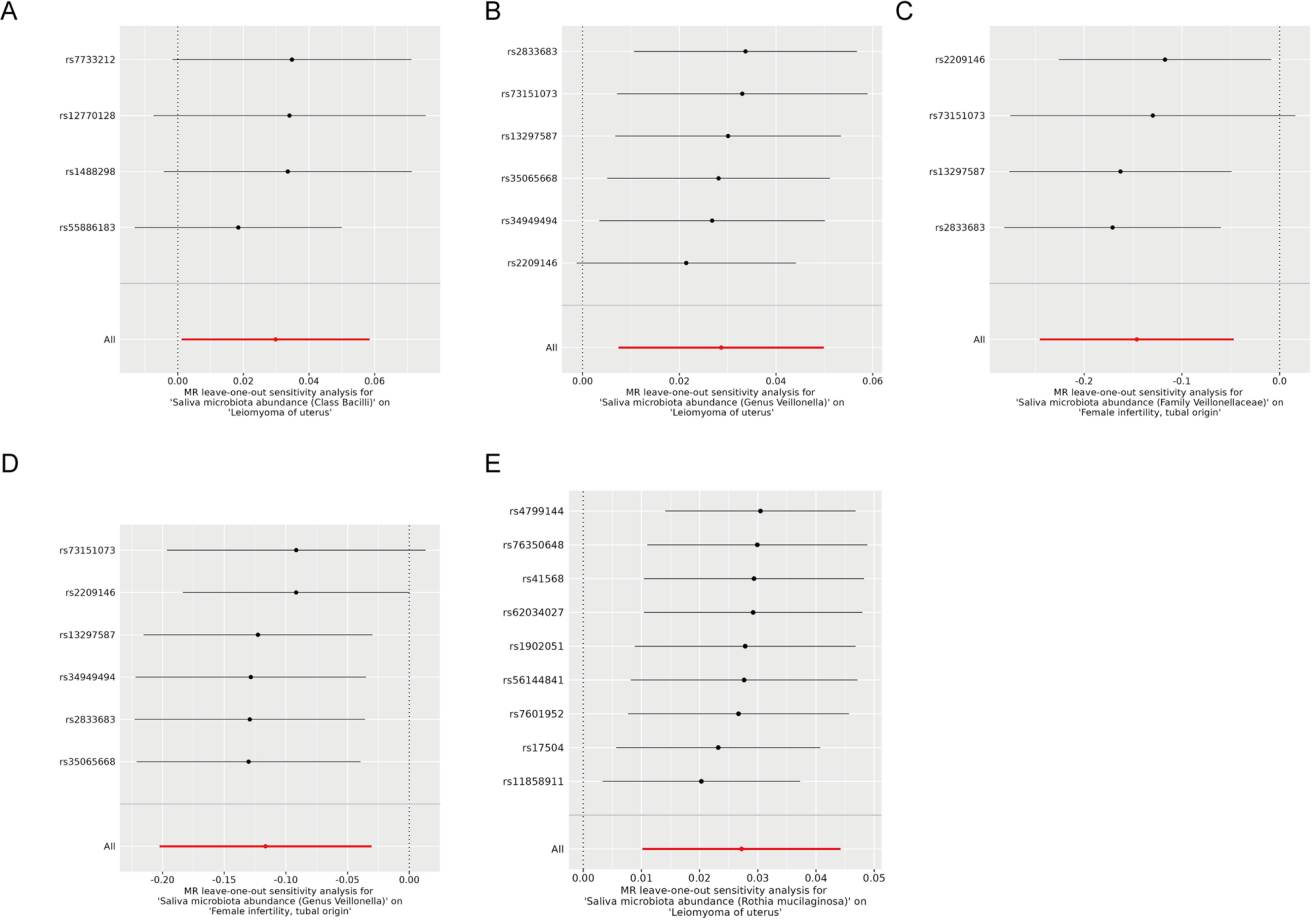
Loo plots of the causal associations between oral microbiota and female reproductive diseases. **A**, **B**: The plots of class *Bacilli* and genus *Veillonella* on uterine leiomyoma; (**C**, **D**): The plots of family *Veillonellaceae* and genus *Veillonella* on female infertility of tubal origin; (**E**): The plot of *Rothia mucilaginosa* on uterine leiomyoma

Overall, sensitivity analyses demonstrated the robustness of the suggestive findings. Funnel plots indicated no observable horizontal pleiotropy (Fig. 4), and leave-one-out sensitivity analyses confirmed that no single SNP disproportionately drove the causal associations (Fig. 5). Finally, post-hoc power calculations indicated that the statistical power to detect these modest causal effects was relatively limited (ranging from 57.94% to 71.40%) (Table S9), which likely explains the loss of statistical significance following rigorous FDR correction.

## Discussion

In this two-sample MR study, we evaluated the causal effects of genetically predicted oral microbiome traits on six female reproductive diseases using publicly available GWAS summary statistics. We identified several nominal IVW associations, primarily involving uterine leiomyoma and tubal infertility; however, none of these signals survived FDR correction. Accordingly, the results should be viewed as exploratory and hypothesis-generating, especially considering the modest exposure GWAS sample size and the multiple-testing burden. In sensitivity analyses, MR-PRESSO outlier correction provided an additional nominal signal for *Rothia mucilaginosa* in relation to uterine leiomyoma; this finding should be interpreted cautiously as exploratory and requires independent confirmation.

### Interpretation of nominal signals

The nominal association between class *Bacilli* and uterine leiomyoma is biologically plausible in so far as oral dysbiosis and periodontal inflammation can contribute to systemic inflammatory tone [5–7]. Importantly, MR estimates effects of genetically predicted microbial traits; it does not demonstrate translocation of live bacteria into reproductive tissues. A more conservative interpretation is that oral dysbiosis may increase circulating microbial components and metabolites (e.g., LPS–TLR4 signaling; SCFAs acting on FFAR2/FFAR3) that modulate immune cell activation and cytokine production [7–9], which are relevant to leiomyoma biology (e.g., uterine smooth muscle cells, fibroblasts, macrophages, and extracellular matrix remodeling) [4]. Similarly, *Veillonellaceae/Veillonella* are common oral anaerobes that may co-vary with oral ecological states linked to inflammation or metabolic profiles [6, 38]; the observed nominal protective association with tubal infertility may reflect host-mediated pathways rather than a direct microbial effect. From a mechanistic perspective, members of class *Bacilli* can produce bioactive metabolites such as polyamines (e.g., spermidine) [39]. *Veillonella* species can generate short-chain fatty acids and other metabolites under specific ecological conditions, and *Veillonella–Lactobacillus* interactions have been reported to modulate intestinal inflammation in experimental models [40, 41]. With respect to *Rothia mucilaginosa*, genome-scale metabolic modeling has characterized its metabolic capabilities, which may inform hypothesis-driven follow-up studies [42]. Although our MR results do not demonstrate translocation of live bacteria into reproductive tissues, microbial translocation has been documented in other clinical contexts involving mucosal barrier injury [43]. More broadly, microbiome–inflammation links are supported by evidence from chronic inflammatory disorders such as inflammatory bowel disease [44].

### Opposite directions for *Veillonella* across outcomes

We observed opposite directions of effect estimates for genus *Veillonella* in tubal infertility versus uterine leiomyoma. Given the small effect sizes, lack of FDR significance, and potential heterogeneity, this pattern should not be over-interpreted as tissue-specific mechanisms. Instead, it may arise from differences in outcome definitions, distinct causal architectures, residual pleiotropy, or statistical fluctuation. Future studies using larger oral microbiome GWAS, colocalization analyses, and functional experiments will be needed to clarify whether this reflects true biological heterogeneity.

### Clinical context of uterine leiomyoma and tubal infertility

Uterine leiomyoma and tubal infertility represent distinct etiologic pathways to infertility: leiomyoma may affect fertility through distortion of the uterine cavity, altered uterine contractility, or endometrial receptivity, whereas tubal infertility is often related to chronic infection, pelvic inflammatory disease, endometriosis, or surgical injury. In many settings, infectious etiologies—particularly pelvic inflammatory disease (including *Chlamydia trachomatis*)—are major contributors to tubal infertility, whereas leiomyoma-related subfertility is more often mediated by uterine anatomy and endometrial receptivity. Therefore, any shared “inflammatory background” should be interpreted as a broad upstream context rather than evidence of a unified disease pathway. Although both conditions may share upstream inflammatory risk factors, our results do not imply a direct clinical link between them. The current findings, if confirmed, would suggest that host genetic determinants of oral microbial traits could be modestly related to specific infertility phenotypes rather than broadly supporting an oral–reproductive axis across FRDs. Notably, the nominal signals were confined to uterine leiomyoma and tubal infertility, while we did not observe consistent evidence across the other FRD outcomes, arguing against a generalized oral–reproductive axis [4, 45].

### Strengths and limitations

The principal strength of this study is the MR framework, which reduces confounding and reverse causation compared with conventional observational studies. We applied multiple sensitivity analyses (MR-Egger intercept, heterogeneity tests, MR-PRESSO, and Steiger directionality) to probe robustness. Several limitations warrant emphasis. First, the oral microbiome GWAS sample size (n = 610) is small, and instrument selection used a relaxed P-value threshold, which may increase the risk of weak-instrument bias and winner’s curse. Such a modest exposure GWAS sample size may limit statistical power and increase the risk of false-negative findings. Therefore, the present results should be interpreted as exploratory and require confirmation in larger oral microbiome GWAS and independent outcome datasets. Second, the exposure GWAS likely included both males and females, whereas FinnGen outcomes were female-specific; sex-related pathways could introduce residual pleiotropy, and female-stratified oral microbiome GWAS are needed. Third, not all predefined oral traits yielded eligible instruments across outcomes, and MR-PRESSO could not be applied to all pairs. Fourth, the effect sizes were small and are not directly applicable for clinical prediction or biomarker development. Furthermore, our analysis was restricted to the 44 microbial traits available in the primary GWAS dataset. Highly specific clinical periodontal pathogens, such as the established ‘red complex’ bacteria (e.g., Porphyromonas gingivalis), were not adequately captured or did not yield robust summary statistics in the original study. Consequently, we were unable to evaluate the specific causal roles of these well-known disease-associated oral microbes [17, 23, 31].

### Future directions

Larger and sex-stratified oral microbiome GWAS, independent replication outcome datasets, and integrative analyses will be valuable to refine causal pathways. Where suitable mediator GWAS are available, multivariable MR and mediation MR (e.g., incorporating inflammatory biomarkers or hormonal traits) could help separate direct microbial-trait effects from host-physiology pathways. Functional studies are needed to evaluate how host genetic determinants of oral microbial traits may influence systemic inflammation and reproductive tissue biology, including the roles of bacterial load, host immunity, and tissue microenvironment [32].

## Conclusions

In conclusion, this two-sample MR analysis provides suggestive genetic evidence that certain oral microbiome traits may be modestly associated with uterine leiomyoma and tubal infertility. Because the associations did not remain significant after FDR correction and effect sizes were small, the findings should be interpreted cautiously and validated in larger, independent studies before any clinical implications are considered.

## Supplementary Information

Supplementary Material 1.

Supplementary Material 2.

Supplementary Material 3.

Supplementary Material 4.

Supplementary Material 5.

Supplementary Material 6.

Supplementary Material 7.

Supplementary Material 8.

Supplementary Material 9.

## Data Availability

The original contributions presented in the study are included in the article/Supplementary Material. Further inquiries can be directed at the corresponding author.

## Abbreviations

CI: Confidence interval
FRDs: Female reproductive diseases
GWAS: Genome-wide association study
HDAC: Histone deacetylase
IVs: Instrumental variables
IVW: Inverse-variance weighted
LD: Linkage disequilibrium
MAF: Minor allele frequency
MR: Mendelian randomization
OR: Odds ratio
PCOS: Polycystic ovary syndrome
SCFAs: Short-chain fatty acids
SNPs: Single nucleotide polymorphisms
STROBE-MR: Strengthening the Reporting of Observational Studies in Epidemiology using Mendelian Randomization

## References

[1] Ma Y, Wu F, Yu Z, Yang L. Evaluating the association between lipidome and female reproductive diseases through comprehensive Mendelian randomization analyses. Sci Rep. 2025;15(1):2448. 39828767 10.1038/s41598-025-86794-2 PMC11743779

[2] Bala R, Singh V, Rajender S, Singh K, Environment. Lifestyle, and Female Infertility. Reprod Sci(Thousand Oaks, Calif). 2021;28(3):617–638. 10.1007/s43032-020-00279-3 32748224

[3] Nestler JE. Polycystic Ovary Syndrome. N Engl J Med. 2016;375(14):1398. 27705266 10.1056/NEJMc1610000

[4] Giuliani E, As-Sanie S, Marsh EE. Epidemiology and management of uterine fibroids. Int J Gynaecol Obstet. 2020;149(1):3–9. 31960950 10.1002/ijgo.13102

[5] Zhang Y, Wang X, Li H, Ni C, Du Z, Yan F. Human oral microbiota and its modulation for oral health. Biomedecine pharmacotherapie. 2018;99:883–93. 29710488 10.1016/j.biopha.2018.01.146

[6] Peng X, Cheng L, You Y, Tang C, Ren B, Li Y, Xu X, Zhou X. Oral microbiota in human systematic diseases. Int J Oral Sci. 2022;14(1):14. 35236828 10.1038/s41368-022-00163-7 PMC8891310

[7] Martínez-García MM, Hernández-Lemus E. Periodontal Inflammation and Systemic Diseases: An Overview. Front Physiol. 2021;12:709438. 10.3389/fphys.2021.709438. 34776994 10.3389/fphys.2021.709438 PMC8578868

[8] Liu X, Hao W, Li J, Zhang H, Zheng Y. Regulation of short-chain fatty acids in the immune system. Front Immunol. 2023;14:1186892. 10.3389/fimmu.2023.1186892. 37215145 10.3389/fimmu.2023.1186892 PMC10196242

[9] Kim CH. Complex regulatory effects of gut microbial short-chain fatty acids on immune tolerance and autoimmunity. Cell Mol Immunol. 2023;20:341–50. 10.1038/s41423-023-00987-1. 36854801 10.1038/s41423-023-00987-1 PMC10066346

[10] Alghamdi S. Isolation and identification of the oral bacteria and their characterization for bacteriocin production in the oral cavity. Saudi J Biol Sci. 2022;29(1):318–23. 35002424 10.1016/j.sjbs.2021.08.096 PMC8716906

[11] Song W, Li D, Tao L, Luo Q, Chen L. Solute carrier transporters: the metabolic gatekeepers of immune cells. Acta Pharm Sinica B. 2020;10(1):61–78. 10.1016/j.apsb.2019.12.006 PMC6977534 31993307

[12] Ohashi A, Murayama MA, Miyabe Y, Yudoh K, Miyabe C. Streptococcal infection and autoimmune diseases. Front Immunol. 2024;15:1361123. 38464518 10.3389/fimmu.2024.1361123 PMC10920276

[13] Mora VP, Loaiza RA, Soto JA, Bohmwald K, Kalergis AM. Involvement of trained immunity during autoimmune responses. J Autoimmun. 2023;137:102956. 36526524 10.1016/j.jaut.2022.102956

[14] Pax K, Buduneli N, Alan M, Meric P, Gurlek O, Dabdoub SM, Kumar PS. Placental TLR recognition of salivary and subgingival microbiota is associated with pregnancy complications. Microbiome. 2024;12(1):64. 38532461 10.1186/s40168-024-01761-9 PMC10964645

[15] Deng L, Guan G, Cannon RD, Mei L. Age-related oral microbiota dysbiosis and systemic diseases. Microb Pathog. 2025;205:107717. 40403989 10.1016/j.micpath.2025.107717

[16] Lamont RJ, Koo H, Hajishengallis G. The oral microbiota: dynamic communities and host interactions. Nat Rev Microbiol. 2018;16(12):745–59. 30301974 10.1038/s41579-018-0089-x PMC6278837

[17] Teles F, Collman RG, Mominkhan D, Wang Y. Viruses, periodontitis, and comorbidities. Periodontology. 2000;2022(1):190–206. 10.1111/prd.12435 35244970

[18] Chadchan SB, Naik SK, Popli P, Talwar C, Putluri S, Ambati CR, Lint MA, Kau AL, Stallings CL, Kommagani R. Gut microbiota and microbiota-derived metabolites promotes endometriosis. Cell Death Discov. 2023;9(1):28. 36693853 10.1038/s41420-023-01309-0 PMC9873805

[19] Sinha T, Brushett S, Prins J, Zhernakova A. The maternal gut microbiome during pregnancy and its role in maternal and infant health. Curr Opin Microbiol. 2023;74:102309. 10.1016/j.mib.2023.102309. 37068462 10.1016/j.mib.2023.102309

[20] Sobstyl A, Chałupnik A, Mertowska P, Grywalska E. How do microorganisms influence the development of endometriosis? Participation of genital, intestinal and oral microbiota in metabolic regulation and immunopathogenesis of endometriosis. Int J Mol Sci. 2023;24(13):10920. 10.3390/ijms241310920. 10.3390/ijms241310920 PMC10341671 37446108

[21] Dou Y, Xin J, Zhou P, Tang J, Xie H, Fan W, Zhang Z, Wu D. Bidirectional association between polycystic ovary syndrome and periodontal diseases. Front Endocrinol (Lausanne). 2023;14:1008675. 36755917 10.3389/fendo.2023.1008675 PMC9899846

[22] Guo JZ, Xiao Q, Gao S, Li XQ, Wu QJ, Gong TT. Review of Mendelian Randomization Studies on Ovarian Cancer. Front Oncol. 2021;11:681396. 34458137 10.3389/fonc.2021.681396 PMC8385140

[23] Holmes MV, Ala-Korpela M, Smith GD. Mendelian randomization in cardiometabolic disease: challenges in evaluating causality. Nat reviews Cardiol. 2017;14(10):577–90. 10.1038/nrcardio.2017.78 PMC5600813 28569269

[24] Venkatesh SS, Ferreira T, Benonisdottir S, Rahmioglu N, Becker CM, Granne I, Zondervan KT, Holmes MV, Lindgren CM, Wittemans LBL. Obesity and risk of female reproductive conditions: A Mendelian randomisation study. PLoS Med. 2022;19(2):e1003679. 35104295 10.1371/journal.pmed.1003679 PMC8806071

[25] Skrivankova VW, Richmond RC, Woolf BAR, Yarmolinsky J, Davies NM, Swanson SA, VanderWeele TJ, Higgins JPT, Timpson NJ, Dimou N, et al. Strengthening the Reporting of Observational Studies in Epidemiology Using Mendelian Randomization: The STROBE-MR Statement. JAMA. 2021;326(16):1614–21. 34698778 10.1001/jama.2021.18236

[26] Sanderson E, Glymour MM, Holmes MV, Kang H, Morrison J, Munafò MR, Palmer T, Schooling CM, Wallace C, Zhao Q, et al. Mendelian randomization. Nat Rev Methods Primers. 2022;2:6. 10.1038/s43586-021-00092-5. 37325194 10.1038/s43586-021-00092-5 PMC7614635

[27] Stankevic E, Kern T, Borisevich D, Poulsen CS, Madsen AL, Hansen TH, Jonsson A, Schubert M, Nygaard N, Nielsen T, et al. Genome-wide association study identifies host genetic variants influencing oral microbiota diversity and metabolic health. Sci Rep. 2024;14(1):14738. 38926497 10.1038/s41598-024-65538-8 PMC11208528

[28] Yang J, Lee SH, Goddard ME, Visscher PM. GCTA: a tool for genome-wide complex trait analysis. Am J Hum Genet. 2011;88(1):76–82. 21167468 10.1016/j.ajhg.2010.11.011 PMC3014363

[29] Wan B, Lu L, Lv C. Mendelian randomization study on the causal relationship between leukocyte telomere length and prostate cancer. PLoS ONE. 2023;18(6):e0286219. 37352282 10.1371/journal.pone.0286219 PMC10289467

[30] Palmer TM, Lawlor DA, Harbord RM, Sheehan NA, Tobias JH, Timpson NJ, Davey Smith G, Sterne JA. Using multiple genetic variants as instrumental variables for modifiable risk factors. Stat Methods Med Res. 2012;21(3):223–42. 21216802 10.1177/0962280210394459 PMC3917707

[31] Bowden J, Davey Smith G, Haycock PC, Burgess S. Consistent Estimation in Mendelian Randomization with Some Invalid Instruments Using a Weighted Median Estimator. Genet Epidemiol. 2016;40(4):304–14. 27061298 10.1002/gepi.21965 PMC4849733

[32] Minelli C, Del Greco MF, van der Plaat DA, Bowden J, Sheehan NA, Thompson J. The use of two-sample methods for Mendelian randomization analyses on single large datasets. Int J Epidemiol. 2021;50(5):1651–9. 33899104 10.1093/ije/dyab084 PMC8580269

[33] Brion MJA, Shakhbazov K, Visscher PM. Calculating statistical power in Mendelian randomization studies. Int J Epidemiol. 2013;42(5):1497–501. 10.1093/ije/dyt179. 24159078 10.1093/ije/dyt179 PMC3807619

[34] Kulinskaya E, Dollinger MB. An accurate test for homogeneity of odds ratios based on Cochran’s Q-statistic. BMC Med Res Methodol. 2015;15:49. 26054650 10.1186/s12874-015-0034-x PMC4531442

[35] Bowden J, Del Greco MF, Minelli C, Davey Smith G, Sheehan NA, Thompson JR. Assessing the suitability of summary data for two-sample Mendelian randomization analyses using MR-Egger regression: the role of the I2 statistic. Int J Epidemiol. 2016;45(6):1961–74. 27616674 10.1093/ije/dyw220 PMC5446088

[36] Verbanck M, Chen CY, Neale B, Do R. Detection of widespread horizontal pleiotropy in causal relationships inferred from Mendelian randomization between complex traits and diseases. Nat Genet. 2018;50(5):693–8. 29686387 10.1038/s41588-018-0099-7 PMC6083837

[37] Hemani G, Tilling K, Davey Smith G. Orienting the causal relationship between imprecisely measured traits using GWAS summary data. PLoS Genet. 2017;13(11):e1007081. 10.1371/journal.pgen.1007081. 29149188 10.1371/journal.pgen.1007081 PMC5711033

[38] Giacomini JJ, Torres-Morales J, Dewhirst FE, Borisy GG, Mark Welch JL. Site Specialization of Human Oral Veillonella Species. Microbiol Spectr. 2023;11(1):e0404222. 36695592 10.1128/spectrum.04042-22 PMC9927086

[39] Suzuki H, Fujiwara Y, Thongbhubate K, Maeda M, Kanaori K. Spore-Forming Lactic Acid-Producing Bacterium Bacillus coagulans Synthesizes and Excretes Spermidine into the Extracellular Space. J Agric Food Chem. 2023;71(25):9868–76. 37314369 10.1021/acs.jafc.3c02184

[40] Hung JH, Zhang SM, Huang SL. Nitrate promotes the growth and the production of short-chain fatty acids and tryptophan from commensal anaerobe Veillonella dispar in the lactate-deficient environment by facilitating the catabolism of glutamate and aspartate. Appl Environ Microbiol. 2024;90(8):e0114824. 39082806 10.1128/aem.01148-24 PMC11337843

[41] Li N, Wang H, Zhao H, Wang M, Cai J, Hao Y, Yu J, Jiang Y, Lü X, Liu B. Cooperative interactions between Veillonella ratti and Lactobacillus acidophilus ameliorate DSS-induced ulcerative colitis in mice. Food Funct. 2023;14(23):10475–92. 37934670 10.1039/d3fo03898j

[42] Leonidou N, Ostyn L, Coenye T, Crabbé A, Dräger A. Genome-scale model of Rothia mucilaginosa predicts gene essentialities and reveals metabolic capabilities. Microbiol Spectr. 2024;12(6):e0400623. 38652457 10.1128/spectrum.04006-23 PMC11237427

[43] Kelly MS, Ward DV, Severyn CJ, Arshad M, Heston SM, Jenkins K, Martin PL, McGill L, Stokhuyzen A, Bhattarai SK, et al. Gut Colonization Preceding Mucosal Barrier Injury Bloodstream Infection in Pediatric Hematopoietic Stem Cell Transplantation Recipients. Biol Blood Marrow Transpl. 2019;25(11):2274–80. 10.1016/j.bbmt.2019.07.019 PMC6861666 31326608

[44] Shan Y, Lee M, Chang EB. The Gut Microbiome and Inflammatory Bowel Diseases. Annu Rev Med. 2022;73:455–68. 34555295 10.1146/annurev-med-042320-021020 PMC10012812

[45] Alexiou ZW, den Heijer CDJ, van Bergen J, et al. Reproductive tract complication risks following Chlamydia trachomatis infections: a long-term prospective cohort study from 2008 to 2022. Lancet Reg Health - Europe. 2024;45:101027. 10.1016/j.lanepe.2024.101027. 39247903 10.1016/j.lanepe.2024.101027 PMC11378087

