# Supplementary material for: Causal effects of oral microbiome traits on female reproductive diseases: a two-sample Mendelian randomization study
Source: BMC Womens Health. 2026 May 22;26:359. doi: 10.1186/s12905-026-04547-3 (PMC13371253; doi:10.1186/s12905-026-04547-3)
Supplement: Supplementary file 3 — Supplementary Material 3. [file 12905_2026_4547_MOESM3_ESM.docx]

Table S3. Tests for horizontal pleiotropy and heterogeneity

| **Exposure** | **Outcome** | **Heterogeneity** | | **Pleiotropy** | |
| --- | --- | --- | --- | --- | --- |
|  |  | **Q statistic (IVW)** | ***P* value** | **MR-Egger Intercept** | ***P* value** |
| Saliva microbiota abundance (Class Bacilli) | Endometriosis | 2.796847 | 0.424019188 | 0.013994 | 0.752043 |
| Saliva microbiota abundance (Class Bacilli) | Female infertility, tubal origin | 0.890542 | 0.82770968 | -0.07753 | 0.512498 |
| Saliva microbiota abundance (Class Bacilli) | Habitual aborter | 1.205952 | 0.75157708 | -0.03064 | 0.8615 |
| Saliva microbiota abundance (Class Bacilli) | Leiomyoma of uterus | 2.860183 | 0.413688248 | -0.03287 | 0.306203 |
| Saliva microbiota abundance (Class Bacilli) | Polycystic ovarian syndrome | 4.712464 | 0.194103994 | 0.170335 | 0.21148 |
| Saliva microbiota abundance (Class Bacilli) | Spontaneous abortion | 9.743567 | 0.020876525 | -0.05045 | 0.469251 |
| Saliva microbiota abundance (Family Actinomycetaceae) | Endometriosis | 0.984278 | 0.912170495 | 0.033075 | 0.655034 |
| Saliva microbiota abundance (Family Actinomycetaceae) | Female infertility, tubal origin | 4.41526 | 0.35271405 | 0.267154 | 0.26343 |
| Saliva microbiota abundance (Family Actinomycetaceae) | Habitual aborter | 4.225615 | 0.37633242 | 0.279825 | 0.453783 |
| Saliva microbiota abundance (Family Actinomycetaceae) | Leiomyoma of uterus | 4.895723 | 0.298165275 | 0.016821 | 0.79993 |
| Saliva microbiota abundance (Family Actinomycetaceae) | Polycystic ovarian syndrome | 4.940661 | 0.293440442 | 0.01017 | 0.96885 |
| Saliva microbiota abundance (Family Actinomycetaceae) | Spontaneous abortion | 4.407066 | 0.353709727 | 0.016859 | 0.831661 |
| Saliva microbiota abundance (Family Lachnospiraceae_[XIV]) | Endometriosis | 20.7435 | 0.007860938 | 0.016227 | 0.639415 |
| Saliva microbiota abundance (Family Lachnospiraceae_[XIV]) | Female infertility, tubal origin | 11.83758 | 0.15859919 | 0.138172 | 0.045986 |
| Saliva microbiota abundance (Family Lachnospiraceae_[XIV]) | Habitual aborter | 10.76907 | 0.215130243 | -0.02871 | 0.803369 |
| Saliva microbiota abundance (Family Lachnospiraceae_[XIV]) | Leiomyoma of uterus | 31.98217 | 9.38E-05 | 0.038675 | 0.183477 |
| Saliva microbiota abundance (Family Lachnospiraceae_[XIV]) | Polycystic ovarian syndrome | 6.940624 | 0.543052236 | 0.060301 | 0.30572 |
| Saliva microbiota abundance (Family Lachnospiraceae_[XIV]) | Spontaneous abortion | 6.718176 | 0.567322007 | 0.001393 | 0.939366 |
| Saliva microbiota abundance (Family Pasteurellaceae) | Endometriosis | 10.21011 | 0.250587327 | 0.037468 | 0.234433 |
| Saliva microbiota abundance (Family Pasteurellaceae) | Female infertility, tubal origin | 4.229161 | 0.835878366 | -0.00504 | 0.949792 |
| Saliva microbiota abundance (Family Pasteurellaceae) | Habitual aborter | 7.755424 | 0.45771846 | 0.163817 | 0.221243 |
| Saliva microbiota abundance (Family Pasteurellaceae) | Leiomyoma of uterus | 15.18526 | 0.055641719 | -0.01385 | 0.631903 |
| Saliva microbiota abundance (Family Pasteurellaceae) | Polycystic ovarian syndrome | 19.49027 | 0.012446541 | 0.069236 | 0.584865 |
| Saliva microbiota abundance (Family Pasteurellaceae) | Spontaneous abortion | 10.88159 | 0.208499222 | 0.029639 | 0.321448 |
| Saliva microbiota abundance (Family Prevotellaceae) | Endometriosis | 24.86364 | 0.000802207 | 0.055088 | 0.3047 |
| Saliva microbiota abundance (Family Prevotellaceae) | Female infertility, tubal origin | 6.873971 | 0.442119024 | -0.06699 | 0.425155 |
| Saliva microbiota abundance (Family Prevotellaceae) | Habitual aborter | 8.632817 | 0.280103815 | 0.017501 | 0.90865 |
| Saliva microbiota abundance (Family Prevotellaceae) | Leiomyoma of uterus | 11.7996 | 0.107344845 | 0.011617 | 0.674727 |
| Saliva microbiota abundance (Family Prevotellaceae) | Polycystic ovarian syndrome | 3.575316 | 0.82718491 | -0.02883 | 0.710695 |
| Saliva microbiota abundance (Family Prevotellaceae) | Spontaneous abortion | 9.482949 | 0.219815449 | 0.006394 | 0.838671 |
| Saliva microbiota abundance (Family Veillonellaceae) | Endometriosis | 3.098525 | 0.376682548 | -0.01432 | 0.538077 |
| Saliva microbiota abundance (Family Veillonellaceae) | Female infertility, tubal origin | 2.560496 | 0.464457156 | -0.00466 | 0.943135 |
| Saliva microbiota abundance (Family Veillonellaceae) | Habitual aborter | 1.029775 | 0.794047783 | -0.03939 | 0.673708 |
| Saliva microbiota abundance (Family Veillonellaceae) | Leiomyoma of uterus | 4.015653 | 0.259778829 | 0.010185 | 0.598039 |
| Saliva microbiota abundance (Family Veillonellaceae) | Polycystic ovarian syndrome | 0.482647 | 0.922686968 | 0.031023 | 0.59246 |
| Saliva microbiota abundance (Family Veillonellaceae) | Spontaneous abortion | 0.970757 | 0.808327493 | -0.0068 | 0.71061 |
| Saliva microbiota abundance (Genus Alloprevotella) | Endometriosis | 0.791987 | 0.373500092 | / | / |
| Saliva microbiota abundance (Genus Alloprevotella) | Female infertility, tubal origin | 0.262332 | 0.608522326 | / | / |
| Saliva microbiota abundance (Genus Alloprevotella) | Habitual aborter | 1.393917 | 0.23774472 | / | / |
| Saliva microbiota abundance (Genus Alloprevotella) | Leiomyoma of uterus | 0.022358 | 0.881139232 | / | / |
| Saliva microbiota abundance (Genus Alloprevotella) | Polycystic ovarian syndrome | 0.75332 | 0.385427078 | / | / |
| Saliva microbiota abundance (Genus Alloprevotella) | Spontaneous abortion | 0.132712 | 0.715636705 | / | / |
| Saliva microbiota abundance (Genus Fusobacterium) | Endometriosis | 9.891792 | 0.450037597 | 0.025668 | 0.201258 |
| Saliva microbiota abundance (Genus Fusobacterium) | Female infertility, tubal origin | 7.597785 | 0.668058832 | -0.03817 | 0.498631 |
| Saliva microbiota abundance (Genus Fusobacterium) | Habitual aborter | 9.390655 | 0.495473134 | 0.038222 | 0.668269 |
| Saliva microbiota abundance (Genus Fusobacterium) | Leiomyoma of uterus | 7.747709 | 0.653464941 | -0.01763 | 0.220274 |
| Saliva microbiota abundance (Genus Fusobacterium) | Polycystic ovarian syndrome | 9.905505 | 0.448822432 | -0.12865 | 0.035344 |
| Saliva microbiota abundance (Genus Fusobacterium) | Spontaneous abortion | 6.339642 | 0.785966602 | -0.01544 | 0.382772 |
| Saliva microbiota abundance (Genus Granulicatella) | Endometriosis | 3.519702 | 0.620408607 | 0.029292 | 0.288218 |
| Saliva microbiota abundance (Genus Granulicatella) | Female infertility, tubal origin | 4.480889 | 0.482444463 | -0.01194 | 0.878809 |
| Saliva microbiota abundance (Genus Granulicatella) | Habitual aborter | 6.303625 | 0.277785743 | -0.06951 | 0.629936 |
| Saliva microbiota abundance (Genus Granulicatella) | Leiomyoma of uterus | 4.8351 | 0.436334635 | 0.003549 | 0.859659 |
| Saliva microbiota abundance (Genus Granulicatella) | Polycystic ovarian syndrome | 2.008057 | 0.848029035 | -0.05776 | 0.435774 |
| Saliva microbiota abundance (Genus Granulicatella) | Spontaneous abortion | 2.003801 | 0.848618824 | -0.00381 | 0.868587 |
| Saliva microbiota abundance (Genus Haemophilus) | Endometriosis | 7.421127 | 0.283649765 | 0.040963 | 0.21646 |
| Saliva microbiota abundance (Genus Haemophilus) | Female infertility, tubal origin | 3.823497 | 0.700547689 | -0.01774 | 0.836853 |
| Saliva microbiota abundance (Genus Haemophilus) | Habitual aborter | 18.40977 | 0.005285749 | 0.272373 | 0.25571 |
| Saliva microbiota abundance (Genus Haemophilus) | Leiomyoma of uterus | 11.72551 | 0.068380128 | -0.03996 | 0.17508 |
| Saliva microbiota abundance (Genus Haemophilus) | Polycystic ovarian syndrome | 11.71461 | 0.068646793 | 0.140447 | 0.228774 |
| Saliva microbiota abundance (Genus Haemophilus) | Spontaneous abortion | 5.46074 | 0.486214441 | 0.011429 | 0.6794 |
| Saliva microbiota abundance (Genus Leptotrichia) | Endometriosis | 0.548887 | 0.759994996 | 0.015568 | 0.658594 |
| Saliva microbiota abundance (Genus Leptotrichia) | Female infertility, tubal origin | 0.760517 | 0.683684793 | 0.060676 | 0.571599 |
| Saliva microbiota abundance (Genus Leptotrichia) | Habitual aborter | 0.753236 | 0.686178248 | 0.028075 | 0.852926 |
| Saliva microbiota abundance (Genus Leptotrichia) | Leiomyoma of uterus | 2.017143 | 0.364739686 | -0.01893 | 0.500326 |
| Saliva microbiota abundance (Genus Leptotrichia) | Polycystic ovarian syndrome | 0.604656 | 0.739095535 | 0.056295 | 0.581481 |
| Saliva microbiota abundance (Genus Leptotrichia) | Spontaneous abortion | 0.000549 | 0.99972544 | 0.000486 | 0.986897 |
| Saliva microbiota abundance (Genus Neisseria) | Endometriosis | 3.129611 | 0.536374345 | 0.042744 | 0.548039 |
| Saliva microbiota abundance (Genus Neisseria) | Female infertility, tubal origin | 1.881196 | 0.757598416 | -0.03851 | 0.847885 |
| Saliva microbiota abundance (Genus Neisseria) | Habitual aborter | 2.671357 | 0.614236051 | 0.324183 | 0.345594 |
| Saliva microbiota abundance (Genus Neisseria) | Leiomyoma of uterus | 0.828003 | 0.934654986 | 0.030723 | 0.547842 |
| Saliva microbiota abundance (Genus Neisseria) | Polycystic ovarian syndrome | 8.089275 | 0.088362143 | -0.2452 | 0.404314 |
| Saliva microbiota abundance (Genus Neisseria) | Spontaneous abortion | 4.226784 | 0.376183183 | 0.032741 | 0.650471 |
| Saliva microbiota abundance (Genus Porphyromonas) | Endometriosis | 1.393172 | 0.707135958 | -0.0439 | 0.428702 |
| Saliva microbiota abundance (Genus Porphyromonas) | Female infertility, tubal origin | 4.415033 | 0.219995529 | -0.0992 | 0.636608 |
| Saliva microbiota abundance (Genus Porphyromonas) | Habitual aborter | 1.09634 | 0.777957602 | -0.04236 | 0.855345 |
| Saliva microbiota abundance (Genus Porphyromonas) | Leiomyoma of uterus | 0.886117 | 0.828776672 | 0.016146 | 0.664537 |
| Saliva microbiota abundance (Genus Porphyromonas) | Polycystic ovarian syndrome | 3.534758 | 0.316282085 | -0.03426 | 0.853983 |
| Saliva microbiota abundance (Genus Porphyromonas) | Spontaneous abortion | 0.337309 | 0.952865426 | -0.01198 | 0.794688 |
| Saliva microbiota abundance (Genus Prevotella) | Endometriosis | 26.08245 | 0.003630859 | 0.021693 | 0.4475 |
| Saliva microbiota abundance (Genus Prevotella) | Female infertility, tubal origin | 18.28385 | 0.050360145 | -0.1283 | 0.041683 |
| Saliva microbiota abundance (Genus Prevotella) | Habitual aborter | 11.81756 | 0.297452375 | -0.08787 | 0.314234 |
| Saliva microbiota abundance (Genus Prevotella) | Leiomyoma of uterus | 13.70404 | 0.186924253 | -0.00381 | 0.801457 |
| Saliva microbiota abundance (Genus Prevotella) | Polycystic ovarian syndrome | 7.814654 | 0.646935576 | -0.00433 | 0.92753 |
| Saliva microbiota abundance (Genus Prevotella) | Spontaneous abortion | 13.63906 | 0.190100904 | -0.0244 | 0.175686 |
| Saliva microbiota abundance (Genus Rothia) | Endometriosis | 4.786714 | 0.442460629 | -0.02647 | 0.251443 |
| Saliva microbiota abundance (Genus Rothia) | Female infertility, tubal origin | 1.803174 | 0.875653739 | 0.038528 | 0.539612 |
| Saliva microbiota abundance (Genus Rothia) | Habitual aborter | 1.555539 | 0.90657305 | -0.07081 | 0.477769 |
| Saliva microbiota abundance (Genus Rothia) | Leiomyoma of uterus | 6.920831 | 0.226595094 | 0.001074 | 0.956862 |
| Saliva microbiota abundance (Genus Rothia) | Polycystic ovarian syndrome | 3.320635 | 0.650684034 | -0.07955 | 0.223053 |
| Saliva microbiota abundance (Genus Rothia) | Spontaneous abortion | 6.964794 | 0.223271821 | 0.013959 | 0.568755 |
| Saliva microbiota abundance (Genus Schaalia) | Endometriosis | 14.32352 | 0.215606014 | -0.00057 | 0.976795 |
| Saliva microbiota abundance (Genus Schaalia) | Female infertility, tubal origin | 13.02604 | 0.291626722 | 0.06487 | 0.210313 |
| Saliva microbiota abundance (Genus Schaalia) | Habitual aborter | 8.173438 | 0.697688372 | 0.036969 | 0.62156 |
| Saliva microbiota abundance (Genus Schaalia) | Leiomyoma of uterus | 16.92701 | 0.110053183 | 0.00797 | 0.59656 |
| Saliva microbiota abundance (Genus Schaalia) | Polycystic ovarian syndrome | 11.54551 | 0.398755655 | 0.002894 | 0.952594 |
| Saliva microbiota abundance (Genus Schaalia) | Spontaneous abortion | 10.02287 | 0.528331089 | 0.006104 | 0.678897 |
| Saliva microbiota abundance (Genus Streptococcus) | Endometriosis | 2.497764 | 0.114008053 | / | / |
| Saliva microbiota abundance (Genus Streptococcus) | Female infertility, tubal origin | 0.009391 | 0.92280001 | / | / |
| Saliva microbiota abundance (Genus Streptococcus) | Habitual aborter | 0.138135 | 0.710142071 | / | / |
| Saliva microbiota abundance (Genus Streptococcus) | Leiomyoma of uterus | 1.870378 | 0.171431807 | / | / |
| Saliva microbiota abundance (Genus Streptococcus) | Polycystic ovarian syndrome | 0.215703 | 0.642333527 | / | / |
| Saliva microbiota abundance (Genus Streptococcus) | Spontaneous abortion | 1.291408 | 0.2557887 | / | / |
| Saliva microbiota abundance (Genus Veillonella) | Endometriosis | 4.622929 | 0.463606297 | -0.01716 | 0.380179 |
| Saliva microbiota abundance (Genus Veillonella) | Female infertility, tubal origin | 4.045716 | 0.542852867 | 0.005558 | 0.918145 |
| Saliva microbiota abundance (Genus Veillonella) | Habitual aborter | 5.448473 | 0.363629056 | -0.01747 | 0.859577 |
| Saliva microbiota abundance (Genus Veillonella) | Leiomyoma of uterus | 4.25042 | 0.513953408 | 0.010637 | 0.442608 |
| Saliva microbiota abundance (Genus Veillonella) | Polycystic ovarian syndrome | 5.805315 | 0.325625976 | 0.012373 | 0.842003 |
| Saliva microbiota abundance (Genus Veillonella) | Spontaneous abortion | 1.346619 | 0.930062306 | -0.00835 | 0.623557 |
| Saliva microbiota abundance (Order Actinomycetales) | Endometriosis | 6.7366 | 0.565299833 | -0.03491 | 0.107058 |
| Saliva microbiota abundance (Order Actinomycetales) | Female infertility, tubal origin | 5.548872 | 0.697619631 | -0.03117 | 0.58832 |
| Saliva microbiota abundance (Order Actinomycetales) | Habitual aborter | 2.130847 | 0.97673539 | -0.0372 | 0.679421 |
| Saliva microbiota abundance (Order Actinomycetales) | Leiomyoma of uterus | 5.188127 | 0.7372927 | 0.007012 | 0.621307 |
| Saliva microbiota abundance (Order Actinomycetales) | Polycystic ovarian syndrome | 5.674773 | 0.683609162 | -0.09743 | 0.107755 |
| Saliva microbiota abundance (Order Actinomycetales) | Spontaneous abortion | 10.28308 | 0.24571656 | 0.02817 | 0.155733 |
| Saliva microbiota abundance (Order Bacteroidales) | Endometriosis | 14.56714 | 0.265965512 | -0.03457 | 0.064026 |
| Saliva microbiota abundance (Order Bacteroidales) | Female infertility, tubal origin | 6.456708 | 0.891334412 | 0.008822 | 0.86017 |
| Saliva microbiota abundance (Order Bacteroidales) | Habitual aborter | 7.618112 | 0.814216697 | 0.038313 | 0.628826 |
| Saliva microbiota abundance (Order Bacteroidales) | Leiomyoma of uterus | 13.30027 | 0.347598976 | 0.016613 | 0.202467 |
| Saliva microbiota abundance (Order Bacteroidales) | Polycystic ovarian syndrome | 9.97841 | 0.61785485 | -0.08129 | 0.111167 |
| Saliva microbiota abundance (Order Bacteroidales) | Spontaneous abortion | 6.158729 | 0.907869944 | 0.006274 | 0.687109 |
| Saliva microbiota abundance (Order Clostridiales) | Endometriosis | 24.48155 | 0.001902061 | 0.046543 | 0.137732 |
| Saliva microbiota abundance (Order Clostridiales) | Female infertility, tubal origin | 8.405418 | 0.394901944 | 0.076092 | 0.182199 |
| Saliva microbiota abundance (Order Clostridiales) | Habitual aborter | 9.746252 | 0.283289337 | -0.02553 | 0.796458 |
| Saliva microbiota abundance (Order Clostridiales) | Leiomyoma of uterus | 32.2742 | 8.32E-05 | 0.028904 | 0.282006 |
| Saliva microbiota abundance (Order Clostridiales) | Polycystic ovarian syndrome | 6.95419 | 0.541583208 | 0.002914 | 0.954347 |
| Saliva microbiota abundance (Order Clostridiales) | Spontaneous abortion | 6.767279 | 0.561937132 | -0.0008 | 0.961497 |
| Saliva microbiota abundance (Order Fusobacteriales) | Endometriosis | 3.851779 | 0.696726542 | -0.00979 | 0.677547 |
| Saliva microbiota abundance (Order Fusobacteriales) | Female infertility, tubal origin | 1.454037 | 0.96248906 | 0.039718 | 0.565725 |
| Saliva microbiota abundance (Order Fusobacteriales) | Habitual aborter | 12.03379 | 0.061219333 | -0.18309 | 0.233572 |
| Saliva microbiota abundance (Order Fusobacteriales) | Leiomyoma of uterus | 3.221404 | 0.780589149 | 0.007286 | 0.666975 |
| Saliva microbiota abundance (Order Fusobacteriales) | Polycystic ovarian syndrome | 5.909833 | 0.433366194 | -0.13878 | 0.075602 |
| Saliva microbiota abundance (Order Fusobacteriales) | Spontaneous abortion | 5.549375 | 0.475509218 | -0.01373 | 0.527822 |
| Saliva microbiota abundance (Phylum Firmicutes) | Endometriosis | 6.081845 | 0.530225894 | -0.01312 | 0.567484 |
| Saliva microbiota abundance (Phylum Firmicutes) | Female infertility, tubal origin | 11.41833 | 0.121384182 | 0.031701 | 0.724627 |
| Saliva microbiota abundance (Phylum Firmicutes) | Habitual aborter | 7.719248 | 0.358000128 | 0.050926 | 0.662675 |
| Saliva microbiota abundance (Phylum Firmicutes) | Leiomyoma of uterus | 12.90446 | 0.074470679 | 0.017528 | 0.450033 |
| Saliva microbiota abundance (Phylum Firmicutes) | Polycystic ovarian syndrome | 15.06658 | 0.035155415 | 0.183463 | 0.023142 |
| Saliva microbiota abundance (Phylum Firmicutes) | Spontaneous abortion | 6.210992 | 0.515340452 | 0.004048 | 0.845451 |
| Saliva microbiota abundance (Phylum Proteobacteria) | Endometriosis | 9.386805 | 0.152964455 | 0.003309 | 0.929097 |
| Saliva microbiota abundance (Phylum Proteobacteria) | Female infertility, tubal origin | 2.813361 | 0.83188101 | 0.004395 | 0.955695 |
| Saliva microbiota abundance (Phylum Proteobacteria) | Habitual aborter | 7.727314 | 0.258769395 | 0.156952 | 0.280585 |
| Saliva microbiota abundance (Phylum Proteobacteria) | Leiomyoma of uterus | 6.848177 | 0.335116314 | 0.02275 | 0.289217 |
| Saliva microbiota abundance (Phylum Proteobacteria) | Polycystic ovarian syndrome | 5.925804 | 0.431552802 | -0.00402 | 0.961203 |
| Saliva microbiota abundance (Phylum Proteobacteria) | Spontaneous abortion | 2.491059 | 0.869466958 | 0.025467 | 0.325502 |
| Saliva microbiota abundance (Species dispar) | Endometriosis | 1.18463 | 0.756693053 | -0.02148 | 0.443945 |
| Saliva microbiota abundance (Species dispar) | Female infertility, tubal origin | 1.580209 | 0.663885676 | -0.02066 | 0.784015 |
| Saliva microbiota abundance (Species dispar) | Habitual aborter | 3.884548 | 0.274203729 | -0.15529 | 0.273578 |
| Saliva microbiota abundance (Species dispar) | Leiomyoma of uterus | 1.303399 | 0.728326094 | -0.0035 | 0.84997 |
| Saliva microbiota abundance (Species dispar) | Polycystic ovarian syndrome | 4.905691 | 0.178834602 | 0.1328 | 0.171117 |
| Saliva microbiota abundance (Species dispar) | Spontaneous abortion | 1.199286 | 0.753175641 | -0.01551 | 0.528897 |
| Saliva microbiota abundance (Species histicola) | Endometriosis | 6.136227 | 0.408104287 | -0.01806 | 0.680957 |
| Saliva microbiota abundance (Species histicola) | Female infertility, tubal origin | 6.994118 | 0.32139152 | -0.08008 | 0.554032 |
| Saliva microbiota abundance (Species histicola) | Habitual aborter | 4.127463 | 0.659431943 | -0.13183 | 0.48547 |
| Saliva microbiota abundance (Species histicola) | Leiomyoma of uterus | 7.013379 | 0.319611684 | 0.000189 | 0.99559 |
| Saliva microbiota abundance (Species histicola) | Polycystic ovarian syndrome | 10.6938 | 0.098313583 | 0.086213 | 0.592957 |
| Saliva microbiota abundance (Species histicola) | Spontaneous abortion | 7.841487 | 0.249947974 | -0.01597 | 0.723566 |
| Saliva microbiota abundance (Species micronuciformis) | Endometriosis | 0.968849 | 0.965039009 | 0.017276 | 0.629659 |
| Saliva microbiota abundance (Species micronuciformis) | Female infertility, tubal origin | 22.24789 | 0.000469723 | 0.310507 | 0.130836 |
| Saliva microbiota abundance (Species micronuciformis) | Habitual aborter | 3.96104 | 0.555038744 | 0.168076 | 0.33081 |
| Saliva microbiota abundance (Species micronuciformis) | Leiomyoma of uterus | 6.732587 | 0.241299758 | 0.010948 | 0.736909 |
| Saliva microbiota abundance (Species micronuciformis) | Polycystic ovarian syndrome | 2.903704 | 0.714828895 | 0.093507 | 0.369432 |
| Saliva microbiota abundance (Species micronuciformis) | Spontaneous abortion | 2.926504 | 0.711315751 | -0.02929 | 0.383221 |
| Saliva microbiota abundance (Species mucilaginosa) | Endometriosis | 6.520641 | 0.769791024 | 0.014283 | 0.37944 |
| Saliva microbiota abundance (Species mucilaginosa) | Female infertility, tubal origin | 14.59149 | 0.147680408 | 0.0045 | 0.93909 |
| Saliva microbiota abundance (Species mucilaginosa) | Habitual aborter | 14.53552 | 0.149935701 | 0.091307 | 0.310344 |
| Saliva microbiota abundance (Species mucilaginosa) | Leiomyoma of uterus | 19.94338 | 0.029792771 | 0.032373 | 0.029151 |
| Saliva microbiota abundance (Species mucilaginosa) | Polycystic ovarian syndrome | 8.604014 | 0.570050235 | -0.04162 | 0.360854 |
| Saliva microbiota abundance (Species mucilaginosa) | Spontaneous abortion | 9.058782 | 0.526534185 | 0.004587 | 0.749984 |
| Saliva microbiota abundance (Species pallens) | Endometriosis | 4.389869 | 0.111365869 | 0.095355 | 0.296019 |
| Saliva microbiota abundance (Species pallens) | Female infertility, tubal origin | 3.873188 | 0.144194206 | 0.215777 | 0.426649 |
| Saliva microbiota abundance (Species pallens) | Habitual aborter | 2.550167 | 0.279407648 | 0.286999 | 0.41525 |
| Saliva microbiota abundance (Species pallens) | Leiomyoma of uterus | 2.673865 | 0.262650159 | 0.051316 | 0.373129 |
| Saliva microbiota abundance (Species pallens) | Polycystic ovarian syndrome | 11.02814 | 0.004029669 | 0.233565 | 0.646278 |
| Saliva microbiota abundance (Species pallens) | Spontaneous abortion | 6.741181 | 0.034369331 | 0.109412 | 0.234531 |
| Saliva microbiota abundance (Species parainfluenzae) | Endometriosis | 1.045758 | 0.592811453 | 0.042883 | 0.537513 |
| Saliva microbiota abundance (Species parainfluenzae) | Female infertility, tubal origin | 1.939366 | 0.379203194 | 0.181055 | 0.420195 |
| Saliva microbiota abundance (Species parainfluenzae) | Habitual aborter | 2.416592 | 0.29870581 | 0.143995 | 0.72615 |
| Saliva microbiota abundance (Species parainfluenzae) | Leiomyoma of uterus | 7.301519 | 0.025971392 | -0.0635 | 0.526141 |
| Saliva microbiota abundance (Species parainfluenzae) | Polycystic ovarian syndrome | 1.934431 | 0.380140039 | 0.118145 | 0.566309 |
| Saliva microbiota abundance (Species parainfluenzae) | Spontaneous abortion | 1.277427 | 0.527971202 | -0.0204 | 0.728582 |
| Saliva microbiota abundance (Species periodonticum) | Endometriosis | 3.151735 | 0.789575772 | 0.039998 | 0.170421 |
| Saliva microbiota abundance (Species periodonticum) | Female infertility, tubal origin | 9.776266 | 0.134395672 | -0.00147 | 0.989005 |
| Saliva microbiota abundance (Species periodonticum) | Habitual aborter | 7.127393 | 0.309226529 | 0.107222 | 0.440279 |
| Saliva microbiota abundance (Species periodonticum) | Leiomyoma of uterus | 0.564531 | 0.996962321 | -0.00496 | 0.793425 |
| Saliva microbiota abundance (Species periodonticum) | Polycystic ovarian syndrome | 4.170587 | 0.653603612 | -0.0823 | 0.290371 |
| Saliva microbiota abundance (Species periodonticum) | Spontaneous abortion | 4.065727 | 0.667782041 | 0.00241 | 0.919168 |
| Saliva microbiota abundance (Species rogosae) | Endometriosis | 0.663457 | 0.881763765 | 0.000174 | 0.996353 |
| Saliva microbiota abundance (Species rogosae) | Female infertility, tubal origin | 1.872153 | 0.599360899 | -0.11376 | 0.365876 |
| Saliva microbiota abundance (Species rogosae) | Habitual aborter | 10.98019 | 0.011833505 | -0.3708 | 0.226691 |
| Saliva microbiota abundance (Species rogosae) | Leiomyoma of uterus | 4.211644 | 0.239498644 | -0.00977 | 0.803262 |
| Saliva microbiota abundance (Species rogosae) | Polycystic ovarian syndrome | 2.348457 | 0.503301459 | -0.10738 | 0.370984 |
| Saliva microbiota abundance (Species rogosae) | Spontaneous abortion | 1.315195 | 0.725528186 | -0.00161 | 0.962564 |
| Saliva microbiota abundance (unknown Gemella) | Endometriosis | 8.38318 | 0.136344028 | -0.02625 | 0.363743 |
| Saliva microbiota abundance (unknown Gemella) | Female infertility, tubal origin | 3.119819 | 0.681519316 | -0.00166 | 0.978439 |
| Saliva microbiota abundance (unknown Gemella) | Habitual aborter | 6.242063 | 0.283372653 | 0.124647 | 0.262353 |
| Saliva microbiota abundance (unknown Gemella) | Leiomyoma of uterus | 2.072886 | 0.838970788 | -0.00983 | 0.529062 |
| Saliva microbiota abundance (unknown Gemella) | Polycystic ovarian syndrome | 4.986597 | 0.41751815 | -0.08934 | 0.18091 |
| Saliva microbiota abundance (unknown Gemella) | Spontaneous abortion | 14.18082 | 0.014500733 | -0.02113 | 0.545439 |
| Saliva microbiota abundance (unknown Neisseria species (ASV0004)) | Endometriosis | 1.757316 | 0.624267289 | 0.021894 | 0.675947 |
| Saliva microbiota abundance (unknown Neisseria species (ASV0004)) | Female infertility, tubal origin | 1.942399 | 0.584447575 | 0.073065 | 0.634503 |
| Saliva microbiota abundance (unknown Neisseria species (ASV0004)) | Habitual aborter | 5.233235 | 0.155493796 | 0.097098 | 0.795837 |
| Saliva microbiota abundance (unknown Neisseria species (ASV0004)) | Leiomyoma of uterus | 3.866969 | 0.276191979 | 0.040858 | 0.359413 |
| Saliva microbiota abundance (unknown Neisseria species (ASV0004)) | Polycystic ovarian syndrome | 2.977574 | 0.395095781 | -0.1625 | 0.326615 |
| Saliva microbiota abundance (unknown Neisseria species (ASV0004)) | Spontaneous abortion | 2.800465 | 0.423423322 | -0.01631 | 0.761606 |
| Saliva microbiota abundance (unknown Porphyromonas species (ASV0008)) | Endometriosis | 3.67116 | 0.29923229 | -0.02667 | 0.648801 |
| Saliva microbiota abundance (unknown Porphyromonas species (ASV0008)) | Female infertility, tubal origin | 2.14557 | 0.542748756 | -0.08701 | 0.529952 |
| Saliva microbiota abundance (unknown Porphyromonas species (ASV0008)) | Habitual aborter | 0.824982 | 0.84348269 | -0.09798 | 0.644814 |
| Saliva microbiota abundance (unknown Porphyromonas species (ASV0008)) | Leiomyoma of uterus | 6.266061 | 0.099359383 | -0.0306 | 0.568833 |
| Saliva microbiota abundance (unknown Porphyromonas species (ASV0008)) | Polycystic ovarian syndrome | 1.469467 | 0.68933515 | -0.10485 | 0.444228 |
| Saliva microbiota abundance (unknown Porphyromonas species (ASV0008)) | Spontaneous abortion | 5.292809 | 0.151569685 | -0.07439 | 0.17309 |
| Saliva microbiota abundance (unknown Rothia species (ASV0012)) | Endometriosis | 0.001081 | 0.973774845 | / | / |
| Saliva microbiota abundance (unknown Rothia species (ASV0012)) | Female infertility, tubal origin | 0.06478 | 0.799094117 | / | / |
| Saliva microbiota abundance (unknown Rothia species (ASV0012)) | Habitual aborter | 0.083819 | 0.772187339 | / | / |
| Saliva microbiota abundance (unknown Rothia species (ASV0012)) | Leiomyoma of uterus | 4.477355 | 0.034346845 | / | / |
| Saliva microbiota abundance (unknown Rothia species (ASV0012)) | Polycystic ovarian syndrome | 0.279897 | 0.596769002 | / | / |
| Saliva microbiota abundance (unknown Rothia species (ASV0012)) | Spontaneous abortion | 1.78997 | 0.180929814 | / | / |
| Saliva microbiota abundance (unknown Rothia species (ASV0016)) | Endometriosis | 5.286049 | 0.381976768 | -0.01922 | 0.471106 |
| Saliva microbiota abundance (unknown Rothia species (ASV0016)) | Female infertility, tubal origin | 4.77615 | 0.443805477 | -0.0941 | 0.22616 |
| Saliva microbiota abundance (unknown Rothia species (ASV0016)) | Habitual aborter | 4.453412 | 0.48614086 | -0.0072 | 0.950706 |
| Saliva microbiota abundance (unknown Rothia species (ASV0016)) | Leiomyoma of uterus | 2.162515 | 0.826231698 | 0.010067 | 0.569266 |
| Saliva microbiota abundance (unknown Rothia species (ASV0016)) | Polycystic ovarian syndrome | 4.229453 | 0.516875347 | -0.0393 | 0.568152 |
| Saliva microbiota abundance (unknown Rothia species (ASV0016)) | Spontaneous abortion | 5.929173 | 0.313171842 | -0.0034 | 0.898005 |
| Saliva microbiota abundance (unknown Schaalia species (ASV0017)) | Endometriosis | 8.782404 | 0.186190381 | 0.035658 | 0.426295 |
| Saliva microbiota abundance (unknown Schaalia species (ASV0017)) | Female infertility, tubal origin | 3.151557 | 0.789598658 | 0.060894 | 0.557252 |
| Saliva microbiota abundance (unknown Schaalia species (ASV0017)) | Habitual aborter | 2.972808 | 0.812251857 | 0.06646 | 0.682584 |
| Saliva microbiota abundance (unknown Schaalia species (ASV0017)) | Leiomyoma of uterus | 10.67326 | 0.099014934 | 0.003274 | 0.92904 |
| Saliva microbiota abundance (unknown Schaalia species (ASV0017)) | Polycystic ovarian syndrome | 5.355522 | 0.499087825 | 0.013226 | 0.895814 |
| Saliva microbiota abundance (unknown Schaalia species (ASV0017)) | Spontaneous abortion | 2.786984 | 0.83506824 | -0.01529 | 0.633909 |
| Saliva microbiota abundance (unknown Streptococcus species (ASV0006)) | Endometriosis | 5.571637 | 0.472841013 | 0.035281 | 0.305786 |
| Saliva microbiota abundance (unknown Streptococcus species (ASV0006)) | Female infertility, tubal origin | 5.892023 | 0.435393917 | -0.02258 | 0.825609 |
| Saliva microbiota abundance (unknown Streptococcus species (ASV0006)) | Habitual aborter | 14.29527 | 0.026506187 | -0.41121 | 0.040364 |
| Saliva microbiota abundance (unknown Streptococcus species (ASV0006)) | Leiomyoma of uterus | 22.49539 | 0.000984355 | 0.040729 | 0.392234 |
| Saliva microbiota abundance (unknown Streptococcus species (ASV0006)) | Polycystic ovarian syndrome | 8.045082 | 0.23481905 | 0.033743 | 0.769086 |
| Saliva microbiota abundance (unknown Streptococcus species (ASV0006)) | Spontaneous abortion | 3.688778 | 0.718707484 | -0.03523 | 0.264106 |
| Saliva microbiota abundance (unknown Streptococcus species (ASV0009)) | Endometriosis | 0.214759 | 0.994631363 | 0.000509 | 0.987685 |
| Saliva microbiota abundance (unknown Streptococcus species (ASV0009)) | Female infertility, tubal origin | 5.202175 | 0.267174901 | -0.04587 | 0.712837 |
| Saliva microbiota abundance (unknown Streptococcus species (ASV0009)) | Habitual aborter | 1.610471 | 0.806908964 | 0.060376 | 0.695218 |
| Saliva microbiota abundance (unknown Streptococcus species (ASV0009)) | Leiomyoma of uterus | 1.567972 | 0.814536702 | 0.019471 | 0.436036 |
| Saliva microbiota abundance (unknown Streptococcus species (ASV0009)) | Polycystic ovarian syndrome | 7.039915 | 0.133793874 | -0.05807 | 0.66493 |
| Saliva microbiota abundance (unknown Streptococcus species (ASV0009)) | Spontaneous abortion | 5.061081 | 0.281087416 | 0.00947 | 0.805367 |
| Saliva microbiota abundance (unknown Veillonella species (ASV0001)) | Endometriosis | 3.785545 | 0.435806462 | -0.02502 | 0.266083 |
| Saliva microbiota abundance (unknown Veillonella species (ASV0001)) | Female infertility, tubal origin | 5.493046 | 0.240341401 | -0.10734 | 0.137418 |
| Saliva microbiota abundance (unknown Veillonella species (ASV0001)) | Habitual aborter | 0.195712 | 0.995513295 | -0.01014 | 0.911048 |
| Saliva microbiota abundance (unknown Veillonella species (ASV0001)) | Leiomyoma of uterus | 2.961892 | 0.564222793 | 0.012127 | 0.424594 |
| Saliva microbiota abundance (unknown Veillonella species (ASV0001)) | Polycystic ovarian syndrome | 5.774205 | 0.216657302 | -0.02711 | 0.720817 |
| Saliva microbiota abundance (unknown Veillonella species (ASV0001)) | Spontaneous abortion | 3.030789 | 0.552686273 | -0.01201 | 0.519618 |
