# Supplementary material for: Causal effects of oral microbiome traits on female reproductive diseases: a two-sample Mendelian randomization study
Source: BMC Womens Health. 2026 May 22;26:359. doi: 10.1186/s12905-026-04547-3 (PMC13371253; doi:10.1186/s12905-026-04547-3)
Supplement: Supplementary file 5 — Supplementary Material 5. [file 12905_2026_4547_MOESM5_ESM.docx]

**Table S5 The eliminated MR-PRESSO results of oral microbiota on FRDs**

| **Exposure** | **Outcome** | **Raw** | |  | **Outlier corrected** | | **Global P** | **Number of outliers** | **Distortion P** |
| --- | --- | --- | --- | --- | --- | --- | --- | --- | --- |
|  |  | **OR (CI%)** | ***P*** |  | **OR (CI%)** | ***P*** |  |  |  |
| Saliva microbiota abundance (Family Lachnospiraceae_[XIV]) | Endometriosis | 0.9816 ( 0.9623 - 1.0013 ) | 0.11022769 |  | NA ( NA - NA ) | NA | 0.814857143 | NA | NA |
| Saliva microbiota abundance (Family Lachnospiraceae_[XIV]) | Female infertility, tubal origin | 1.0925 ( 1.0161 - 1.1748 ) | 0.053986644 |  | NA ( NA - NA ) | NA | 0.739142857 | NA | NA |
| Saliva microbiota abundance (Family Lachnospiraceae_[XIV]) | Leiomyoma of uterus | 1.0103 ( 0.989 - 1.032 ) | 0.382943161 |  | NA ( NA - NA ) | NA | 0.396857143 | NA | NA |
| Saliva microbiota abundance (Family Prevotellaceae) | Endometriosis | 0.9967 ( 0.9657 - 1.0286 ) | 0.842218902 |  | NA ( NA - NA ) | NA | 0.275142857 | NA | NA |
| Saliva microbiota abundance (Genus Fusobacterium) | Polycystic ovarian syndrome | 1.0027 ( 0.9588 - 1.0486 ) | 0.908323189 |  | NA ( NA - NA ) | NA | 0.873285714 | NA | NA |
| Saliva microbiota abundance (Genus Haemophilus) | Habitual aborter | 0.9971 ( 0.8609 - 1.1549 ) | 0.970754258 |  | NA ( NA - NA ) | NA | 0.339142857 | NA | NA |
| Saliva microbiota abundance (Genus Prevotella) | Endometriosis | 0.9939 ( 0.9698 - 1.0186 ) | 0.637669616 |  | NA ( NA - NA ) | NA | 0.430714286 | NA | NA |
| Saliva microbiota abundance (Genus Prevotella) | Female infertility, tubal origin | 1.0389 ( 0.9632 - 1.1206 ) | 0.348559066 |  | NA ( NA - NA ) | NA | 0.357857143 | NA | NA |
| Saliva microbiota abundance (Order Clostridiales) | Endometriosis | 0.9882 ( 0.96 - 1.0173 ) | 0.449034057 |  | NA ( NA - NA ) | NA | 0.397142857 | NA | NA |
| Saliva microbiota abundance (Order Clostridiales) | Leiomyoma of uterus | 1.0121 ( 0.9904 - 1.0342 ) | 0.320282517 |  | NA ( NA - NA ) | NA | 0.426714286 | NA | NA |
| Saliva microbiota abundance (Phylum Firmicutes) | Habitual aborter | 1.0012 ( 0.8761 - 1.1443 ) | 0.985900909 |  | NA ( NA - NA ) | NA | 0.373285714 | NA | NA |
| Saliva microbiota abundance (Phylum Firmicutes) | Polycystic ovarian syndrome | 1.0252 ( 0.9307 - 1.1293 ) | 0.629348405 |  | NA ( NA - NA ) | NA | 0.15 | NA | NA |
| Saliva microbiota abundance (Species micronuciformis) | Female infertility, tubal origin | 0.9702 ( 0.9141 - 1.0298 ) | 0.392989753 |  | NA ( NA - NA ) | NA | 0.789857143 | NA | NA |
| Saliva microbiota abundance (Species mucilaginosa) | Leiomyoma of uterus | 1.0276 ( 1.0102 - 1.0452 ) | 0.013942012 |  | NA ( NA - NA ) | NA | 0.356571429 | NA | NA |
| Saliva microbiota abundance (unknown Gemella) | Spontaneous abortion | 0.9989 ( 0.9645 - 1.0345 ) | 0.952571456 |  | NA ( NA - NA ) | NA | 0.329142857 | NA | NA |
| Saliva microbiota abundance (unknown Streptococcus species (ASV0006)) | Habitual aborter | 0.9977 ( 0.8499 - 1.1711 ) | 0.97843754 |  | NA ( NA - NA ) | NA | 0.437 | NA | NA |
| Saliva microbiota abundance (unknown Streptococcus species (ASV0006)) | Leiomyoma of uterus | 0.9815 ( 0.953 - 1.011 ) | 0.283848823 |  | NA ( NA - NA ) | NA | 0.268 | NA | NA |
