# Supplementary material for: Causal effects of oral microbiome traits on female reproductive diseases: a two-sample Mendelian randomization study
Source: BMC Womens Health. 2026 May 22;26:359. doi: 10.1186/s12905-026-04547-3 (PMC13371253; doi:10.1186/s12905-026-04547-3)
Supplement: Supplementary file 7 — Supplementary Material 7. [file 12905_2026_4547_MOESM7_ESM.docx]

**Table S7.** The eliminated results for horizontal pleiotropy and heterogeneity

| **Exposure** | **Outcome** | **Heterogeneity** | |  | **Pleiotropy** | |
| --- | --- | --- | --- | --- | --- | --- |
|  |  | **Q statistic (IVW)** | ***P* value** |  | **MR-Egger Intercept** | ***P* value** |
| Saliva microbiota abundance (Family Lachnospiraceae_[XIV]) | Endometriosis | 3.691414 | 0.814554954 |  | 0.01752 | 0.405449 |
| Saliva microbiota abundance (Family Lachnospiraceae_[XIV]) | Female infertility, tubal origin | 3.661868 | 0.722324073 |  | 0.07522 | 0.337654 |
| Saliva microbiota abundance (Family Lachnospiraceae_[XIV]) | Leiomyoma of uterus | 6.303374 | 0.390077925 |  | 0.020372 | 0.297038 |
| Saliva microbiota abundance (Family Prevotellaceae) | Endometriosis | 7.791431 | 0.253785323 |  | 0.029978 | 0.383838 |
| Saliva microbiota abundance (Genus Fusobacterium) | Polycystic ovarian syndrome | 4.671257 | 0.861972843 |  | -0.09007 | 0.193071 |
| Saliva microbiota abundance (Genus Haemophilus) | Habitual aborter | 6.163608 | 0.290623202 |  | 0.128347 | 0.459805 |
| Saliva microbiota abundance (Genus Prevotella) | Endometriosis | 9.12909 | 0.425444502 |  | 0.001027 | 0.957345 |
| Saliva microbiota abundance (Genus Prevotella) | Female infertility, tubal origin | 10.15637 | 0.337975 |  | -0.08069 | 0.181859 |
| Saliva microbiota abundance (Order Clostridiales) | Endometriosis | 7.639699 | 0.365433798 |  | 0.022311 | 0.29444 |
| Saliva microbiota abundance (Order Clostridiales) | Leiomyoma of uterus | 6.180382 | 0.403290153 |  | -0.00093 | 0.955191 |
| Saliva microbiota abundance (Phylum Firmicutes) | Habitual aborter | 7.719248 | 0.358000128 |  | 0.050926 | 0.662675 |
| Saliva microbiota abundance (Phylum Firmicutes) | Polycystic ovarian syndrome | 8.724796 | 0.189657107 |  | 0.142989 | 0.095474 |
| Saliva microbiota abundance (Species micronuciformis) | Female infertility, tubal origin | 0.946423 | 0.814212951 |  | 0.11818 | 0.653146 |
| Saliva microbiota abundance (Species mucilaginosa) | Leiomyoma of uterus | 9.318409 | 0.316151488 |  | 0.029496 | 0.065052 |
| Saliva microbiota abundance (unknown Gemella) | Spontaneous abortion | 5.019451 | 0.285307552 |  | -0.01335 | 0.588862 |
| Saliva microbiota abundance (unknown Streptococcus species (ASV0006)) | Habitual aborter | 5.4934 | 0.35867013 |  | -0.20021 | 0.578429 |
| Saliva microbiota abundance (unknown Streptococcus species (ASV0006)) | Leiomyoma of uterus | 5.593083 | 0.231667779 |  | 0.04453 | 0.145 |
